# Supplementary material for: Multisite, mixed methods study to validate 10 maternal health system and policy indicators in Argentina, Ghana and India: a research protocol
Source: BMJ Open. 2022 Jan 17;12(1):e049685. doi: 10.1136/bmjopen-2021-049685 (PMC8765031; doi:10.1136/bmjopen-2021-049685)
Supplement: Author's manuscript [file bmjopen-2021-049685.draft_revisions.pdf]

BMJ Open is committed to open peer review. As part of this commitment we make the peer review history of every article we publish publicly available.

When an article is published we post the peer reviewers' comments and the authors' responses online. We also post the versions of the paper that were used during peer review. These are the versions that the peer review comments apply to.

The versions of the paper that follow are the versions that were submitted during the peer review process. They are not the versions of record or the final published versions. They should not be cited or distributed as the published version of this manuscript.

BMJ Open is an open access journal and the full, final, typeset and author-corrected version of record of the manuscript is available on our site with no access controls, subscription charges or pay-per-view fees (http://bmjopen.bmj.com).

If you have any questions on BMJ Open's open peer review process please email info.bmjopen@bmj.com

BMJ Open

A multisite, mixed methods study to validate ten maternal health system and policy indicators in Argentina, Ghana, and India: a research protocol

Journal:	*BMJ Open*
Manuscript ID	bmjopen-2021-049685
Article Type:	Protocol
Date Submitted by the Author:	22-Mar-2021
Complete List of Authors:	Jolivet, R.; Harvard University T H Chan School of Public Health, Global Health & Population<br>Gausman, Jewel; Harvard University T H Chan School of Public Health, Women and Health Initiative; Department of Global Health and Population<br>Adanu, Richard; University of Ghana School of Public Health,<br>Bandoh, Delia; University of Ghana School of Public Health<br>Belizan, M; Institute for Clinical Effectiveness and Health Policy<br>Bergel, Eduardo; Institute for Clinical Effectiveness and Health Policy<br>Berrueta, Mabel; Institute for Clinical Effectiveness and Health Policy, Data Center<br>Chakraborty, Suchandrima; Population Council<br>Kenu, Ernest; University of Ghana School of Public Health<br>Khan, Nizamuddin; Population Council India,<br>Odikro, Magdalene; University of Ghana<br>Pingray, Veronica; Institute for Clinical Effectiveness and Health Policy<br>Ramesh, Sowmya<br>Saggurti, Niranjan; Population Council<br>Vasquez, Paula; Institute for Clinical Effectiveness and Health Policy<br>Langer , Ana ; Harvard University Department of Global Health and Population
Keywords:	Health policy < HEALTH SERVICES ADMINISTRATION & MANAGEMENT, International health services < HEALTH SERVICES ADMINISTRATION & MANAGEMENT, OBSTETRICS, GYNAECOLOGY, PUBLIC HEALTH, REPRODUCTIVE MEDICINE

SCHOLARONE™ Manuscripts

Title: A multisite, mixed methods study to validate ten maternal health system and policy indicators in Argentina, Ghana, and India: a research protocol

Authors: R. Rima Jolivet, Jewel Gausman, Richard Adanu, Delia Bandoh, Maria Belizan, Eduardo Bergel, Mabel Berrueta, Suchandrima Chakraborty, Ernest Kenu, Nizamuddin Khan, Magdalene Odikro, Veronica Pingray, Sowmya Ramesh, Niranjan Saggurti, Paula Vazquez, Ana Langer

1. Women and Health Initiative, Department of Global Health and Population, Harvard T.H. Chan School of Public Health, Boston, MA, USA
2. School of Public Health, University of Ghana, Legon, Accra,Ghana
3. Institute for Clinical Effectiveness and Health Policy (IECS), Buenos Aires, Argentina
4. Population Council, Inc., New Delhi, India

Corresponding Author:
R. Rima Jolivet, CNM, MSN, DrPH
Department of Global Health and Population
Harvard T.H. Chan School of Public Health
651 Huntington Avenue, FXB 643C, Boston, MA 02115
rjolivet@hsph.harvard.edu

Notes:

- The dates of the study are May 2020 – October 2021.

DRAFT

For peer review only

ABSTRACT [296/300]

Introduction

Most efforts to assess maternal health indicator validity focus on measures of service coverage. Fewer measures focus on the upstream enabling environment, and such measures are typically not research-validated. Thus, methods for validating system and policy-level indicators are not well described. This protocol describes original multi-country research to be conducted in Argentina, Ghana, and India, to validate ten indicators from the monitoring framework for the "Strategies toward Ending Preventable Maternal Mortality" (EPMM). The overall aim is to improve capacity to drive and track progress towards achieving the priority recommendations in the EPMM Strategies. This work is expected to contribute new knowledge on validation methodology, and reveal important information about the indicators under study and the phenomena they target for monitoring. Validating the indicators in three diverse settings will explore the external validity of results.

Methods and analysis

This observational study explores the validity of ten indicators from the EPMM monitoring framework via seven discrete validation exercises that will utilize mixed methods: 1) cross-sectional review of policy data, 2) retrospective review of facility-level patient and administrative data, and 3) collection of primary quantitative and qualitative cross-sectional data from health service providers and clients. There is a specific methodological approach and analytic plan for each indicator, directed by unique, relevant validation research questions.

Ethics and dissemination

The protocol was approved by the Office of Human Research Administration at Harvard University in November, 2019. Individual study sites received approval via local institutional review boards by January 2020 except La Pampa, Argentina, approved June 2020.

Our dissemination plan enables unrestricted access and reuse of all published research, including data sets. We expect to publish at least one peer-reviewed publication per validation exercise. We will disseminate results at conferences, and engage local stakeholders in dissemination activities in each study country.

For peer review only - http://bmjopen.bmj.com/site/about/guidelines.xhtml

ARTICLE SUMMARY

Strengths and limitations of this study

- Will contribute new knowledge on validation methodology to the field of maternal health measurement
- Will reveal important information about the underlying constructs that the ten discrete indicators under study are intended to measure and the phenomena they target for monitoring
- Testing and validating the selected indicators in twelve districts selected by systematic sampling across three diverse settings will help to explore the external validity of the results
- Will contribute toward improving metrics, data quality, and measurement capacity to monitor distal determinants of maternal mortality that comprise an enabling environment for maternal health and survival
- Will not reflect comprehensive national data but rather focuses on four subnational study settings in each country

DRAFT
For peer review only

Word Count [4238/4000]

Introduction

Sustainable Development Goal (SDG) 3.1.1. targets a global maternal mortality ratio (MMR) of <70 maternal deaths per 100,000 live births by 2030. There were 295,000 maternal deaths in 2017, a global MMR of 211/100,000. If the average annual rate of reduction does not accelerate above 2.9%, the rate from 2000 to 2017, we will miss the target by 1 million preventable maternal deaths worldwide. (1) As countries move through the obstetric transition (2) and maternal deaths shift from direct obstetric to indirect causes, addressing upstream factors is critical to ending preventable maternal mortality. Graham et al. (2016) (3) illustrated the widening range of causes of death between and within countries. Thus, recognition is growing of the importance of social, political, economic, and structural factors that impact causes of death and health system responses to them. These include the status of women in societies, the functionality of health systems, access to universal health coverage and reproductive justice, the capacity to register all births and to count all deaths and track their causes, and to address all causes effectively. With acknowledgement of the significance of such distal determinants, improving metrics, data quality, and measurement capacity to monitor them has taken on greater urgency.

In 2015, the World Health Organization (WHO) released the "Strategies toward Ending Preventable Maternal Mortality (EPMM)" (EPMM Strategies) (4), a global guidance document outlining targets and strategies for reducing maternal mortality in the SDG period. Developed through extensive stakeholder consultations, the strategies address the broad spectrum of determinants of maternal health and survival, exemplified in 11 Key Themes.

Table 1. EPMM 11 Key Themes

**Guiding Principles**	1. Empower women, girls, families and communities
	2. Integrate maternal and newborn health, protect and support the mother-baby dyad
	3. Prioritize country ownership, leadership, and supportive legal, regulatory and financial frameworks
	4. Apply a human-rights framework to ensure that high-quality reproductive, maternal, and newborn health care is available, accessible and acceptable to all who need it
**Cross-cutting Actions**	5. Improve metrics, measurement systems, and data quality
	6. Prioritize adequate resources and effective health care financing
**Five Strategic Objectives**	7. Address inequities in access to and quality of sexual, reproductive, maternal and newborn healthcare
	8. Ensure universal health coverage for comprehensive sexual, reproductive, maternal, and newborn healthcare
	9. Address all causes of maternal mortality, reproductive and maternal morbidities and related disabilities
	10. Strengthen health systems to respond to the needs and priorities of women and girls
	11. Ensure accountability in order to improve quality of care and equity

For peer review only - http://bmjopen.bmj.com/site/about/guidelines.xhtml

In 2016, over 150 technical, policy, and country experts from 78 organizations worldwide participated in a five-round modified Delphi process to develop a comprehensive monitoring framework for the EPMM Strategies, comprising indicators centered on its 11 Key Themes. A set of 25 indicators, plus six indicator stratification factors to allow tracking of inequities and data transparency, were identified by participants as the strongest available measures for tracking progress toward the priority recommendations in the report. (5) The organizing framework of the EPMM 11 Key Themes and menu of associated indicators were designed to support national decision makers in identifying priority areas for improvement in their context, and in tracking and driving improvement in those areas deemed of greatest relevance and urgency.

In 2019, the WHO "Mother and Newborn Information for Tracking Outcomes and Results" (MoNITOR) expert working group commissioned a landscape analysis based on interviews with experts in maternal and newborn health (MNH) measurement to better understand how the field conceptualizes indicator validity, approaches to validation, and gaps in the science (6).

(Box 1.) What is Indicator Validity?

WHAT DOES INDICATOR VALIDITY MEAN?

Validity asks, "Is this measurement truly representative of the concept under study?"

**Selected Types of Validity**	**Definition**
Content Validity	Does the indicator fully represent the content domain or concept to be measured?
Criterion-related Validity	How does the value of an indicator compare to an objective measure of truth?
Construct Validity	Do two indicators that are purported to measure the same construct "behave" in the same way?

The analysis identified gaps in research on indicator validity conducted in LMIC settings, and poor knowledge translation about indicator validity to those settings. As a result, it found little application of information on validity in the evaluation and selection of indicators for national and subnational monitoring. Some types of indicators in particular lacked research-based validation, e.g. those for monitoring women's satisfaction and experiences of care; abortion services; as well as indicators derived from facility and routine data systems and the policy environment. Recommendations included engaging national stakeholders in discussions and research on indicator validity, and focusing beyond diagnostic-style, criterion-related validity to encompass the meaningfulness of indicators, including the accurate definition of their underlying constructs and their utility to drive improvement.

Most efforts to assess maternal health indicator validity focus on measures of service coverage (7) (8) (9, 10) and, to a lesser extent, quality and reliability of service delivery (11) (6) (12). Fewer measures

For peer review only - http://bmjopen.bmj.com/site/about/guidelines.xhtml

overall focus on the upstream enabling environment for maternal health care provision, and they are typically not subject to validation research. (13) Methods for validation of health system and policy-level indicators are therefore not well described.

Benova et al. (14) (2020) published a conceptual framework compiling definitions of indicator validity and approaches for assessing its various dimensions, based on interviews with practitioners of MNH measurement. The framework includes methodological approaches for assessing validity of indicators for tracking health policy and health system factors, and calls for more research in this domain.

Aiming to fill critical gaps in the assessment of maternal health measure validity, the present protocol describes multi-country research to be conducted in Argentina, Ghana, and India at both national and sub-national levels. The overall aim of the study is to improve maternal health measurement by validating ten indicators from the EPMM framework, which correspond to nine of the 11 Key Themes (Figure 1), in order to drive improvement and track progress towards achieving the priority recommendations outlined in the EPMM Strategies.

(Figure 1.) Ten Indicators for Validation and their Corresponding EPMM Key Themes

Methods and analysis

This observational study explores the validity of ten indicators from the EPMM monitoring framework via seven discrete validation exercises. It utilizes mixed methods, including 1) cross-sectional review of secondary policy, legal, and regulatory data, 2) retrospective review of facility-level patient and administrative data, and 3) collection of primary, quantitative, cross-sectional data from health service providers and clients. There is a specific methodological approach to validate each indicator.

The ten EPMM indicators under study and the specific validation research questions for each indicator appear in Table 2. All validation exercises will be conducted in all countries, except for #7, to be validated in Ghana only, due to local interest. Data collection began in January 2020, was suspended due to COVID-19, resumed May 2020, and is expected to be completed by November 2021 in all settings.

For peer review only - http://bmjopen.bmj.com/site/about/guidelines.xhtml

(Table 2.) Indicators for Validation and Validation Questions

Validation Exercise	Indicators for Validation	Validation Questions
#1:	Legal status of abortion	1. How does the law, as expressed in the national statute, compare to the Countdown indicator metadata and to the information available on the WHO Global Abortion Policies Project Database for the country?<br><br>2. Is there evidence that providers are consistently applying the law for each of the grounds on which abortion is legal?
#2	If fees exist for health services in the public sector, are women of reproductive age (15-49) exempt from user fees for [maternal health -related] services	1. Does the free care law or policy in the country provide all of the categories of services included in the indicator free of charges or fees to users?<br><br>2. For the categories of services that should be free according to the law/policy in the country, is there evidence that women are paying user fees for them?<br><br>3. If evidence is found that demonstrates that women are paying for services that are supposed to be free according to the law/policy in the country, is there evidence that user fees are being levied in a systematically differential way to women?
#3a	Health worker density and distribution (per 1,000 population)<br><br>Density of midwives, by district (by births)	1. How does the definition of a midwife/midwifery professional on record in the country compare to the ILO definition and to the ICM midwifery competencies?<br><br>2. What proportion of practicing midwives meet the ICM standard for competency as evidenced by an analysis of the tasks they have performed in the last 90-day period?<br><br>3. How does the value of the estimate differ based on the denominator used?

For peer review only - http://bmjopen.bmj.com/site/about/guidelines.xhtml

#3b	Midwives are authorized to deliver basic emergency obstetric and newborn care	1. Does the national regulatory framework in country that authorizes midwives/MPs to deliver BEmONC match was has been reported for this indicator for all 7 signal functions?<br><br>2. For signal functions that midwives/MPs are authorized to perform according to national regulations, is there evidence they have performed these tasks in settings where EmONC is provided in last year?
#4	Availability of functional EmOC facilities<br><br>Geographic distribution of facilities that provide basic and EmOC	1. Is there evidence from facilities designated as B/CEmONC to demonstrate that they have performed all 7 signal functions in last 3 months as defined in the metadata for these indicators?<br><br>2.How does the value of the indicator differ based on the denominator used: 500,000 population/district vs. 20,000 birth/district vs. travel time (<2 hours for BEmONC)?
#5	Maternal death review coverage	1. How does evidence from the facility level on maternal death reviews compare to the coverage of maternal death reviews reported at district level, through state or district reporting programs?<br><br>2. How does the number of facility deaths captured through review of facility patient register data compare to the number of deaths reported at the district level?<br><br>3. How does the value of the indicator reported compare to the value calculated using primary data?
#6	Demand for family planning satisfied through modern methods of contraception	1. How does a direct measure of demand satisfaction for family planning (woman's self-report) compare to the assigned result provided by the DHS algorithm derived from the responses to the series of questions used to calculate the indicator (same woman surveyed) (construct validity)?

For peer review only - http://bmjopen.bmj.com/site/about/guidelines.xhtml

		2. How does the value of the indicator vary based on a new data source/estimation method compared to an established source/method?
#7	Presence of laws and regulations that guarantee women aged 15-49 access to sexual and reproductive health care, information, and education	1. Do the laws or regulations as recorded on the national statute in Ghana match the definition of the indicator, fully including all 13 components? (Presence of laws)<br>2. How does the value of the indicator change using two different methods of computation (scoring)?

peer review only
DRAFT

Research Settings
The research will be coordinated by a multi-country team of partners from all three countries and the US. Country partners were selected through a competitive process based on proposal strength and geographic diversity. One application was selected from Africa, Asia, and Latin America/Caribbean respectively, based on World Bank classification. (15)

The research will comprise national and sub-national data; however, fieldwork will be conducted in subnational settings in each country. Four districts/provinces in each country were selected for primary data collection. Sites were selected through a purposive, two-staged sampling approach based on a composite index of key maternal health indicators reflecting antepartum, intrapartum, and postpartum care coverage and MMR, used as a proxy of health system performance. First, one state/region in the highest-performing quartile of the index and one state/region in the lowest-performing quartile were selected. Second, one highest-performing district/province and one lowest-performing district/province were selected within each state/region. Due to low population density in Argentina, terciles were used. In addition, because there was almost no geographic variability in skilled birth attendance and early postnatal care coverage in data from Argentina where most births take place in facilities, Uterotonic Administration at Birth (UAB) was substituted in the index for this country.

(Table 3.) National and Subnational Research Settings

Country	State / Region	District/Province
Argentina	Centro	Buenos Aires
		La Pampa
	Noroeste	Salta
		Jujuy
Ghana	Brong Ahafo	Techiman North
		Sunyani Municipal
	Northern	Bunkpurugu-Yunyoo
		Tolon
India	Tamil Nadu	Thiruvallur
		Krishnagiri
	Uttar Pradesh	Meerut
		Gonda

Data Sources, Participants, and Sampling
Data required for validation vary by indicator; details of the data sources, participants, and sampling for each indicator are presented in Table 4.

For peer review only - http://bmjopen.bmj.com/site/about/guidelines.xhtml

(Table 4.) Data Sources, Participants, and Sampling Plan Detailed by Validation Exercise

Validation Exercise	National/Subnational Data Sources	Facility-Level Data			Individual-Level Data		
		Facility Selection	Facility Sampling Plan	Data Source	Participant Selection	Participant Sampling Plan	Data Source
**#1**	National/subnational document review<br><br>Countdown 2030 country profile<br><br>WHO Global Abortion Policies Project (GAPP) Database	Sample of Facilities within 20 PSUs<br><br>All higher-level facilities in study districts/provinces	All facilities that perform at least one maternal-health related service	No facility-level data collected	All health service providers who belong to professional cadres that are legally authorized to provide abortion within the study setting	All eligible health service providers in all eligible facilities	Survey administered to eligible providers
**#2**	National/subnational document review<br><br>WHO Maternal Newborn Child and Adolescent Health Policy Survey (MNCAH)	Sample of Facilities within 20 PSUs<br><br>All higher-level facilities in study districts/provinces	All facilities that perform at least one maternal-health related service	No facility-level data collected	Chief financial officer (or similar administrative position) for each facility<br><br>Woman who received maternal health-related services<br><br>Companion of choice (e.g. family member or friend, if applicable) for women who had a complicated	All chief financial officers in all eligible facilities<br><br>All eligible women (or their companion of choice) leaving eligible facilities	Interviews with chief financial officers<br><br>Exit interviews with women or their companion of choice

					birth and/or underwent a cesarean		
**#3a**	National/subnational document review<br><br>District/Provincial demographic data including total population, number of women of reproductive age, number of births, and number of pregnancies	Census of all facilities in study districts/provinces	All facilities that perform at least one maternal-health related service	Facility staff listing	All currently employed professionals who meet the International Labour Organization's description of midwifery professionals or midwifery associate professionals	All eligible providers in all eligible facilities (in facilities with more than 50 eligible providers, a random sample of 50 providers will be drawn).	Survey administered to midwifery professional/midwifery associate professionals
**#3b**	National/subnational document review	Sample of Facilities within 20 PSUs<br><br>All higher-level facilities in study districts/provinces	All C/B EmONC facilities	Not applicable	All currently employed professionals who meet the International Labour Organization's description of midwifery professionals or midwifery associate professionals	All eligible providers in all eligible facilities (in facilities with more than 50 eligible providers, a random sample of 50 providers will be drawn).	Survey administered to midwifery professional/midwifery associate professionals
**#4**	District/Provincial demographic data including total population, number of women of reproductive age,	Census of all facilities in study districts/provinces	All facilities that provide birth care in each district/province	Facility GIS locational data	Not Applicable	Not Applicable	Not Applicable

For peer review only - http://bmjopen.bmj.com/site/about/guidelines.xhtml

	number of births, and number of pregnancies						
**#5**	Health Information System Data<br><br>Death Reviews reported to district/province	Census of all facilities in study districts/provinces	All facilities that provide birth care in each district/province	Administrative data<br><br>Registers	Not Applicable	Not Applicable	Not Applicable
**#6**	Not Applicable	Not Applicable	Not Applicable	Not Applicable	Community-based Sample of women*	Women aged between 15 and 49 years in study districts	Individual interview
**#7**	National/subnational document review<br><br>United Nations 12th Inquiry Among Governments on Population and Development, Module II (Fertility, Family Planning, and Reproductive Health) Survey	Not Applicable	Not Applicable	Not Applicable	Not Applicable	Not Applicable	Not Applicable

For peer review only - http://bmjopen.bmj.com/site/about/guidelines.xhtml

In general, three types of data will be collected: policy/administrative, facility, and individual data. We will systematically search for national and subnational policies, laws, and regulations through a comprehensive desk review of relevant source documents in each country. Country research teams will consult with subject matter experts and data custodians to ensure all relevant documents were captured. Country-specific data will also be collected from global databases and repositories, as required by each indicator. Further, administrative and patient-level data will be collected from district/provincial-level health management information systems (HMIS).

Facilities will be selected based on data requirements for each indicator, using a multi-stage sampling plan (Figure 2). In the first stage, we will conduct a census of all public and private registered health facilities in each study district/province. For some indicators, data will be collected from all facilities in the census. Next, we will determine which maternal health-related services are provided at each facility in the census. We will collect information on provision of services within the five categories in the WHO Maternal Newborn Child and Adolescent Health (MNCAH) Policy Survey: 1) cesarean section, childbirth (normal delivery), delivery-related pharmaceutical products and medical supplies, 2) family planning, 3) antenatal care and insecticide treated bed nets, 4) postnatal care for mother, 5) testing and treatment for sexually transmitted infectious diseases, and cervical cancer screening. (16) Although infertility management is included in the WHO MNCAH Policy Survey, it is not in our study.

Thereafter, we will replicate the methodology used in Demographic and Health Surveys (DHS) (17) to define primary sampling units (PSUs), which are typically census tracts or discrete villages, depending on the country. We will randomly select 20 PSUs in each study district/province based on probability proportionate to size. Finally, we will define eligible facilities for each indicator within the sampled PSUs based on the services they provide relevant to the specific validation questions for that indicator. Eligible facilities for each indicator will include all lower-level primary health facilities within the PSUs that provide the relevant maternal health-related services, plus all higher-level facilities across the district/province.

(Figure 2.) Schematic of Standard Sampling Plan for Facilities

Within study districts/provinces, we will collect primary, quantitative, individual-level data from study participants via surveys conducted at facilities and in communities. Eligible facility-based participants will include administrators; maternity care clinicians (midwives/midwifery professionals and clinical cadres legally authorized to provide induced abortions); women who received an included maternal-health related service at an eligible facility, and their chosen companions if they had a complicated childbirth or cesarean birth. Within eligible facilities, we will obtain a sample of staff participants as detailed in Table 4. We will enroll 1,040 women of reproductive age who received maternal health services in each country, representing 20 women per service/district for 260 women total per district.

Eligible community-based participants will include women of reproductive age (15-49 years). We will use the same 20 PSUs to obtain the community-based sample of women. Within each, a house listing exercise will identify households with women of reproductive age (15-49 years). From this list, 18 households per PSU will be randomly selected and 1420 women will be recruited, based on the following sample size calculation: $n = \frac{Z^2 * pq}{d^2}$, where Z is the standard normal deviate, p is the proportion of population with characteristic, q is the proportion of population without characteristic, d is the degree of accuracy required. The sample size derived through this calculation (n=96) was further adjusted to reflect an estimated 10% non-response rate, a design effect of 2 to account for clustering,

For peer review only - http://bmjopen.bmj.com/site/about/guidelines.xhtml

and a multiplier of 1.68 to account for the low prevalence of modern contraception in each country, yielding a final sample size of 355 women per district/province. Household surveys are infeasible in Argentina due to low population density, vast distances between households, and lack of cultural acceptance. Therefore, interviews will be conducted with a random sample of 360 women per district exiting from eligible facilities.

Eligibility and exclusion criteria

Participants will be considered eligible if they belong to one of the targeted participant groups listed above, and/or have received an included maternal health related-service, and meet the age of majority to consent or else provide assent along with parental consent if younger (less than 18 years old in Ghana and India; less than 16 years old in Argentina).

Exclusion criteria include not being proficient in the local language; not meeting the age of majority in the country, district, or province unless they can provide parental consent; being unable, unwilling, or lacking capacity to provide consent or assent.

Public and Patient Involvement

No patients were involved in the design, conducting, reporting, or dissemination of this study. We will engage local country stakeholders in a dissemination activity in each study country. We will disseminate results to district/provincial government units and participating health facilities as appropriate, to ensure that they can be used to drive progress and improvement in the study settings.

In the following section, we describe in detail the specific methodology and analytical plan for each indicator.

Validation Exercise #1: Validating "legal status of abortion" as an indicator of equal access under the law

Aims: 1) To verify that the "legal status of abortion" indicator reported globally by each country accurately reflects the laws and statutes on record; and 2) To look for variation at the provider- and facility-level of the application of the legal categories under which abortion is lawful (legal grounds), and thus the accessibility of induced abortion.

Methods: This validation exercise will use mixed methods exploring two validation questions to test the global indicator on legal status of abortion. We will conduct a desk review of the legal grounds for induced abortion expressed in national laws (subnational laws, in Argentina), also capturing any requirements for eligibility on each legal ground articulated in the legal statutes. We will conduct surveys with health professionals whose scope of practice authorizes them to provide abortions services in each setting to explore provider knowledge of the legal grounds for abortion in their jurisdiction, and provider practices for determining patient eligibility on each legal ground, providing abortion services or referrals.

Analysis: For the first validation question, we will compare and describe any differences between legal statutes in each country, reported data in the Countdown indicator, and the WHO GAPP Database. For the second, we will tabulate the number of accurate survey responses among abortion providers on the legal grounds for abortion in their jurisdiction. We will explore any variance in provider requirements to access abortion for each legal ground in the country to look for differences in the application of the law

For peer review only - http://bmjopen.bmj.com/site/about/guidelines.xhtml

across providers and facilities. Descriptive statistics will be reported and we will stratify the results to look for systematic variance.

Validation Exercise #2: Validating reported policies on free maternal health-related services in the public sector

Aim: To verify that no charges, formal or informal, are assessed for services included in the indicator that are supposed to be free by law, and to describe variance between the law and primary data sources.

Methods: We will conduct a desk review of national and subnational laws and policies on free care provision. We will administer surveys to chief financial officers (or similar administrative position) within participating health facilities to collect data on formal fees or payments charged for any included services and the rationale. We will conduct interviews with women exiting eligible facilities to ask about formal and informal charges for any services received. If a woman had a complicated birth or cesarean section and a companion of choice (e.g. family member or friend) is present who was at the facility during the birth, we will interview them as well about any charges they may have paid on her behalf.

Analysis: We will use comparative analysis to detect and describe differences between service categories designated as free to users in the national statutes, and the most recent data reported by the country in the WHO MNCAH Policy Survey. We will estimate the percent of women paying fees for each type of service. Universal applicability of the policy implies that 0% of women pay fees for maternal health services in the public sector. We will test the significance in the difference using a one-sample test of proportion. We will use a chi-squared test to determine if fees are levied in a systematically different way to various types of women using the EPMM standard equity stratifiers. Results will be reported by service type and client demographics, and the value of the indicator expressed each way will be compared to explore optimal construct validity.

Validation Exercise #3a and #3b: Validating critical measures for monitoring adequacy of the midwifery workforce

Aim: To strengthen measurement of midwifery workforce adequacy. Three aspects of adequacy are reflected: density (number to meet need), distribution (accessibility), and both competency and authorization to provide essential care (availability).

Two nested validation exercises are included. The aims of the first one are: 1) to compare midwifery professionals' scope of practice in each country to international reference standards from the International Labour Organization's (ILO) definitions for midwifery professionals and associate professionals and to the International Confederation of Midwives (ICM) Essential Competencies for Midwifery Practice; and 2) to compare estimates derived from two indicators to measure the same construct (density and distribution of midwives), to explore consistency (convergent validity), evidence that one measure is more accurate or a more efficient way to capture the construct, and whether adjusting the numerator and/or denominator provides a better estimate.

The second validation exercise aims to verify whether midwives and midwifery professionals are authorized to perform basic obstetric and neonatal care (BEmONC) functions, and whether they do so in practice.

For peer review only - http://bmjopen.bmj.com/site/about/guidelines.xhtml

Methods: We will conduct document review to compare the national scope of practice for midwifery professionals on record in each country to the ILO and ICM descriptions for midwifery personnel. We will review national laws and regulations that authorize midwifery professionals' scope of practice in each country to verify what is reported by the country in the MNCAH Policy Survey. Then, we will recruit a representative sample of midwifery professionals employed within all participating facilities providing maternal health-related services in each study district. We will administer a survey asking respondents whether they have the skills necessary to perform each competency and/or BEmONC signal function; how they obtained those skills; the frequency and recency of behaviors related to each competency; or reasons for non-performance of these behaviors in their current job.

Analysis: We will report the percent agreement between the national scope of midwifery practice and the ILO tasks, and the ICM competencies, and the variance between them. We will calculate the percent (%) of midwives whose current practice meets the international standard reflected in the ICM competencies as well as the average competency of midwives in the sample, stratified by facility type (public, private), and geography (urban, rural). Last, we will compare the value of the indicator for density and distribution of midwives, adjusted using different numerators and denominators. For numerators, we will calculate the value using the number of midwives on facility rosters, those who meet the ILO definition, and those who meet the ICM competencies. For the denominator, we will examine the value of the indicator using different population parameters: total population/district; women of reproductive age/district; number of births/district; and number of pregnancies/district.

We will compare midwives' authorization to perform BEmONC signal functions with the country's most recent Countdown 2030 country profile and responses to the most recent WHO RMNCH Policy Survey. We will then compare the tasks that midwives and midwifery professionals are authorized to perform to their reported actual performance of those tasks over the last 90-day period in facilities where emergency maternal and newborn care is available in each study setting. We will report any variance between midwifery professionals' authorization, training, and practice patterns.

Validation Exercise #4: Triangulating measures of availability - Validating indicators for monitoring "Availability of B/CEmoNC facilities"

Aim: To explore two dimensions of availability of B/CEmONC facilities: availability of all B/CEmONC signal functions within designated B/CEmONC facilities, and sufficient number of B/CEmONC facilities to meet the needs of the population (coverage). The aim is to compare the value of estimates emphasizing different dimensions of availability of B/CEmONC facilities, based on different measurement approaches and data sources, to explore their external consistency or convergent validity.

Methods: We will review records at all participating facilities where births take place to look for evidence that they have performed emergency signal functions within the previous 90 days and offer services 24 hours per day/7 days per week. We will perform geospatial analysis to estimate the travel time to each facility within the sample for various segments of the population. We will use a publicly available global population model for these estimations.

Analysis: We will compare and report any variance between B/CEmONC designation and functionality across all facilities. We will calculate and compare the value of the indicator in each study district using the following denominators: 500,000 population/district; 20,000 births/district; 30,000 pregnancies/district. Last, we will use the travel time estimates obtained from the geospatial analysis to

For peer review only - http://bmjopen.bmj.com/site/about/guidelines.xhtml

ascertain the number of facilities that are within a two-hour travel time for the total population, for women of reproductive age, and for the number of births and pregnancies occurring to women within each study district. We will explore how the value of the indicator differs based on the denominator used, and compare the values of the indicator reflecting these various approaches to measuring EmONC availability and report differences.

Validation Exercise #5: Validating "maternal death review coverage" to improve maternal mortality data.

Aim: To validate both numerator and denominator of the indicator "Maternal death review coverage", defined as the percentage of maternal deaths occurring in a facility that were audited, in the study settings. Both numerator and denominator are subject to threats to validity due to under-reporting and misclassification of maternal deaths.

Methods: We will collect documentary evidence of maternal deaths and maternal deaths reviews in all facilities through chart and record review. We will perform retrospective review of secondary data obtained from district HMIS on both maternal deaths and maternal death reviews reported from all facilities.

Analysis: We will compare the number of facility-based maternal deaths reported through HMIS to the district to the verified number of maternal deaths in all facilities in the district in patient registers. We will trace individual deaths by dates and other reported details to verify they have been reported to the district. Once validated, we will aggregate all maternal deaths reported for comparison. We will review facility death review committee records for the last one-year period to extract the number of maternal death reviews conducted and the content of each review. We will compare the number of maternal death reviews reported to each district with the number of reviews validated through facility record review that met the definitional standard for quality (18) in the same district. Finally, we will tabulate maternal death review coverage using primary data for the numerator and denominator to the official value reported in the indicator in each country.

Validation Exercise #6: Validating "Demand for family planning satisfied" from a woman-centered perspective: does the indicator reflect women's lived experience?

Aim: "Demand for family planning satisfied through modern methods of contraception" uses a macroeconomic lens to look at contraceptive supply and demand, aggregating data from individual women; however, it is uncertain how well it correlates with women's own subjective perceptions of their personal demand for contraception through modern methods or how well that demand has been satisfied. This study has two aims: 1) at the individual level, to assess whether women's self-reported demand for family planning and its satisfaction converges with the standard DHS-derived measure, and 2) at the population level, to examine how the value of the indicator changes based on the use of derived data from the standard calculation versus self-reported data reflecting women's own perceptions.

Methods: We will administer a community-based survey to a sample of women in each study setting that includes direct questions to women about their desire for and use of contraception, their satisfaction with their current method, and their experience of care during their most recent family

For peer review only - http://bmjopen.bmj.com/site/about/guidelines.xhtml

planning encounter. We will then ask all the questions, in order, in the DHS algorithm used as the global standard to calculate the indicator.

Analysis: We will compare the results for individual women of two different approaches to measuring the construct of “demand for family planning satisfied through modern methods of contraception” using matched t-tests. We will disaggregate by women’s characteristics to identify patterns. Finally, at the population level, we will compare the value of the indicator calculated from primary data we collect to the aggregate district/province level data reported through DHS where available to explore convergence.

Validation Exercise #7: Comparative analysis of two scoring approaches to SDG 5.6.2. and their impact on the indicator value and interpretation of the results

Aim: Sustainable Development Goal 5.6.2. tracks the “Number of countries with laws and regulations that guarantee full and equal access to women and men aged 15 years and older to sexual and reproductive health care, information and education.” Weaknesses with the indicator scoring methodology have the potential to change its value and affect its interpretation. The aim of this exercise is to verify the laws and regulations reported for this indicator in Ghana, and to explore whether the value of the indicator changes using new estimation methods to calculate its score compared to the established method, to improve interpretation.

Methods: We will conduct a comprehensive desk review of legal statutes and regulations related to the 13 components in the indicator metadata. We will conduct secondary analysis of results from the United Nations Twelfth Inquiry Among Governments on Population and Development, Module II (Fertility, Family Planning, and Reproductive Health) Survey(19), which reports on existing laws along with barriers and enablers.

Analysis: We will compare the laws and regulations on record in Ghana to the 13 components reported in the indicator for completeness and accuracy. We will calculate scores for the data collected from the UN Module II survey using the original UN scoring method and alternative scoring methods to look for differences in resulting values of the indicator. Values will be compared and sensitivity analyses conducted to explore the range of variation in the value of the indicator and the associated impact on its interpretation as a measure of sexual and reproductive health and rights.

For peer review only - http://bmjopen.bmj.com/site/about/guidelines.xhtml

References

1. World Health O. Trends in maternal mortality 2000 to 2017: estimates by WHO, UNICEF, UNFPA, World Bank Group and the United Nations Population Division. Geneva: World Health Organization; 2019 2019.
2. Souza JP, Tunçalp Ö, Vogel J, Bohren M, Widmer M, Oladapo O, et al. Obstetric transition: the pathway towards ending preventable maternal deaths. BJOG: An International Journal of Obstetrics & Gynaecology. 2014;121:1-4.
3. Graham W, Woodd S, Byass P, Filippi V, Gon G, Virgo S. Maternal health 1 diversity and divergence: the dynamic burden of poor maternal health. Lancet. 2016;388:2164-75.
4. Organization WH. Strategies towards ending preventable maternal mortality (EPMM). 2015.
5. Jolivet RR, Moran AC, O'Connor M, Chou D, Bhardwaj N, Newby H, et al. Ending preventable maternal mortality: phase II of a multi-step process to develop a monitoring framework, 2016–2030. BMC pregnancy and childbirth. 2018;18(1):258.
6. Benova L, Moller A-B, Moran AC. "What gets measured better gets done better": The landscape of validation of global maternal and newborn health indicators through key informant interviews. PloS one. 2019;14(11):e0224746.
7. Bryce J, Arnold F, Blanc A, Hancioglu A, Newby H, Requejo J, et al. Measuring coverage in MNCH: new findings, new strategies, and recommendations for action. PLoS Med. 2013;10(5):e1001423.
8. Munos MK, Stanton CK, Bryce J. Improving coverage measurement for reproductive, maternal, neonatal and child health: gaps and opportunities. Journal of global health. 2017;7(1).
9. Carvajal–Aguirre L, Vaz LM, Singh K, Sitrin D, Moran AC, Khan SM, et al. Measuring coverage of essential maternal and newborn care interventions: an unfinished agenda. Journal of global health. 2017;7(2).
10. Moran A, Marchant T. Measuring coverage of essential maternal and newborn care interventions: an unfinished agenda to define the data matrix for action in maternal and newborn health. Journal of global health. 2017;7(2).
11. Saturno-Hernández PJ, Martínez-Nicolás I, Moreno-Zegbe E, Fernández-Elorriaga M, Poblano-Verástegui O. Indicators for monitoring maternal and neonatal quality care: a systematic review. BMC pregnancy and childbirth. 2019;19(1):25.
12. Moller A-B, Newby H, Hanson C, Morgan A, El Arifeen S, Chou D, et al. Measures matter: a scoping review of maternal and newborn indicators. PLoS One. 2018;13(10):e0204763.
13. George A, LeFevre AE, Jacobs T, Kinney M, Buse K, Chopra M, et al. Lenses and levels: the why, what and how of measuring health system drivers of women's, children's and adolescents' health with a governance focus. BMJ Global Health. 2019;4(Suppl 4):e001316.
14. Benova L, Moller A-B, Hill K, Vaz LM, Morgan A, Hanson C, et al. What is meant by validity in maternal and newborn health measurement? A conceptual framework for understanding indicator validation. PloS one. 2020;15(5):e0233969.
15. Bank TW. The World by Income and Region: The World Bank; [Available from: https://datatopics.worldbank.org/world-development-indicators/the-world-by-income-and-region.html.
16. Organization WH. Sexual, reproductive, maternal, newborn, child and adolescent health: policy survey, 2018-2019: summary report. 2020.
17. Corsi DJ, Neuman M, Finlay JE, Subramanian S. Demographic and health surveys: a profile. International journal of epidemiology. 2012;41(6):1602-13.

For peer review only - http://bmjopen.bmj.com/site/about/guidelines.xhtml

18. Organization WH. Time to respond: A report on the global implementation of maternal death surveillance and response. Geneva: WHO; 2016. 2017.
19. Lavrakas P. Encyclopedia of Survey Research Methods. 2008.

Authors contributions

1. Rima Jolivet and Jewel Gausman drafted this paper with inputs from all co-authors.
2. Rima Jolivet developed the proposal for funding with inputs from Ana Langer and colleagues from the EPMM Working Group and the Harvard T.H. Chan School of Public Health.
3. All co-authors collaborated to conceptualize and co-develop the research aims and methods. Jewel Gausman led the development of the analytic plans with review and input from all co-authors.
4. All co-authors provided substantive review feedback to finalize the paper.

Acknowledgements
Lenka Benova
Ann-Beth Moller
Allisyn Moran
Jeff Blossom

Funding statement
This work was supported by the Bill and Melinda Gates Foundation with grant number OPP1169546.

Competing interests statement
None declared.

DRAFT
For peer review only

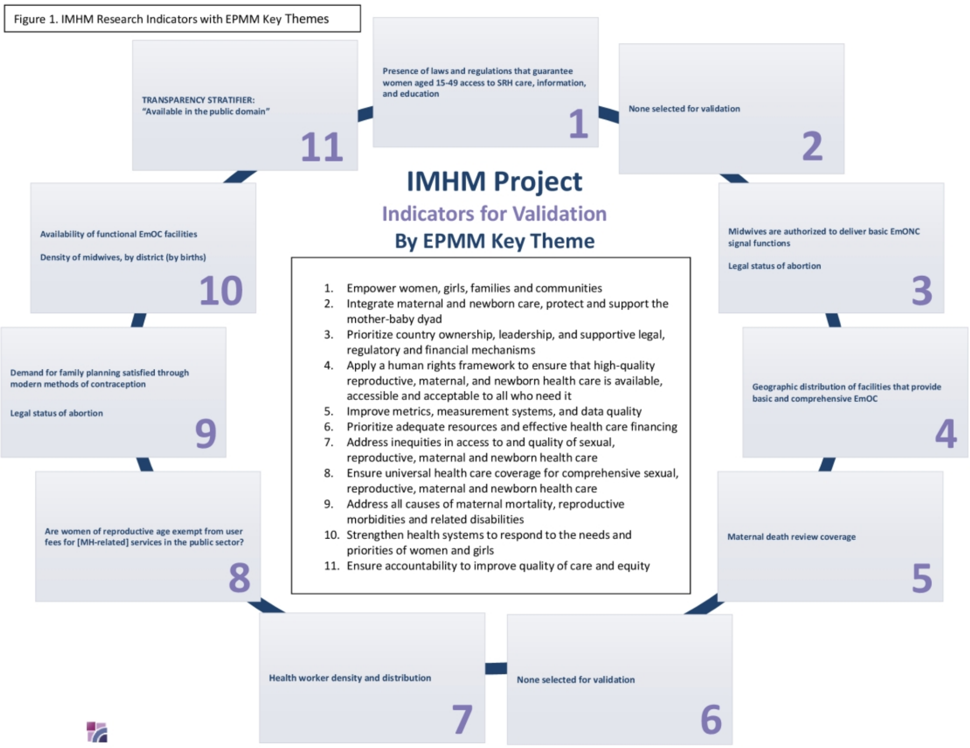

Figure 1. Molecule Diagram - IMHM Research Indicators with EPMM Key Themes

279x215mm (200 x 200 DPI)

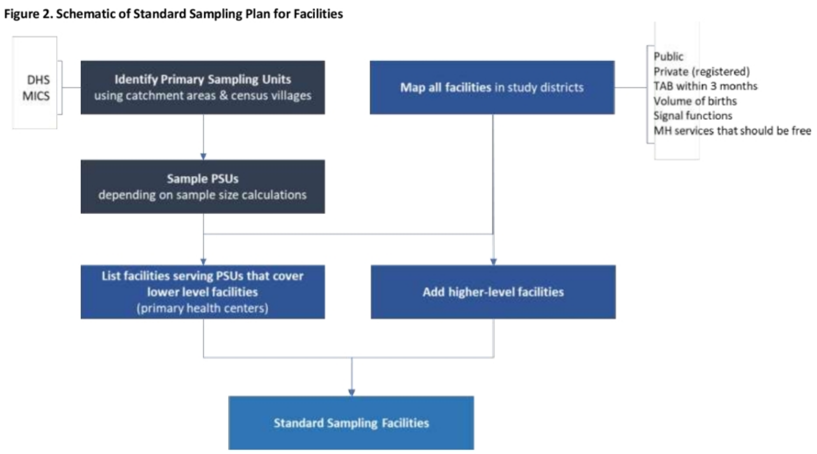

Figure 2. Schematic of Standard Sampling Plan for Facilities

279x215mm (200 x 200 DPI)

BMJ Open

A multisite, mixed methods study to validate ten maternal health system and policy indicators in Argentina, Ghana, and India: a research protocol

Journal:	*BMJ Open*
Manuscript ID	bmjopen-2021-049685.R1
Article Type:	Protocol
Date Submitted by the Author:	01-Oct-2021
Complete List of Authors:	Jolivet, R. Rima; Harvard University T H Chan School of Public Health, Global Health & Population<br>Gausman, Jewel; Harvard University T H Chan School of Public Health, Women and Health Initiative; Department of Global Health and Population<br>Adanu, Richard; University of Ghana School of Public Health,<br>Bandoh, Delia; University of Ghana School of Public Health<br>Belizan, M; Institute for Clinical Effectiveness and Health Policy<br>Bergel, Eduardo; Institute for Clinical Effectiveness and Health Policy<br>Berrueta, Mabel; Institute for Clinical Effectiveness and Health Policy, Data Center<br>Chakraborty, Suchandrima; Population Council<br>Kenu, Ernest; University of Ghana School of Public Health<br>Khan, Nizamuddin; Population Council India,<br>Odikro, Magdalene; University of Ghana<br>Pingray, Veronica; Institute for Clinical Effectiveness and Health Policy<br>Ramesh, Sowmya<br>Saggurti, Niranjan; Population Council<br>Vázquez, Paula; Institute for Clinical Effectiveness and Health Policy<br>Langer , Ana ; Harvard University Department of Global Health and Population
**Primary Subject Heading**:	Global health
Secondary Subject Heading:	Obstetrics and gynaecology, Public health, Health services research, Health policy
Keywords:	Health policy < HEALTH SERVICES ADMINISTRATION & MANAGEMENT, International health services < HEALTH SERVICES ADMINISTRATION & MANAGEMENT, OBSTETRICS, GYNAECOLOGY, PUBLIC HEALTH, REPRODUCTIVE MEDICINE

SCHOLARONE™
Manuscripts

Title: A multisite, mixed methods study to validate ten maternal health system and policy indicators in Argentina, Ghana, and India: a research protocol

Authors: R. Rima Jolivet, Jewel Gausman, Richard Adanu, Delia Bandoh, Maria Belizan, Eduardo Bergel, Mabel Berrueta, Suchandrima Chakraborty, Ernest Kenu, Nizamuddin Khan, Magdalene Odikro, Veronica Pingray, Sowmya Ramesh, Niranjan Saggurti, Paula Vázquez, Ana Langer

1. Women and Health Initiative, Department of Global Health and Population, Harvard T.H. Chan School of Public Health, Boston, MA, USA
2. School of Public Health, University of Ghana, Legon, Accra,Ghana
3. Institute for Clinical Effectiveness and Health Policy (IECS), Buenos Aires, Argentina
4. Population Council, Inc., New Delhi, India

Corresponding Author:
R. Rima Jolivet, CNM, MSN, DrPH
Department of Global Health and Population
Harvard T.H. Chan School of Public Health
651 Huntington Avenue, FXB 643C, Boston, MA 02115
rjolivet@hsph.harvard.edu

Notes:

- The dates of the study are May 2020 – November 2021.

For peer review only - http://bmjopen.bmj.com/site/about/guidelines.xhtml

ABSTRACT [296/300]

Introduction

Most efforts to assess maternal health indicator validity focus on measures of service coverage. Fewer measures focus on the upstream enabling environment, and such measures are typically not research-validated. Thus, methods for validating system and policy-level indicators are not well described. This protocol describes original multi-country research to be conducted in Argentina, Ghana, and India, to validate ten indicators from the monitoring framework for the "Strategies toward Ending Preventable Maternal Mortality" (EPMM). The overall aim is to improve capacity to drive and track progress towards achieving the priority recommendations in the EPMM Strategies. This work is expected to contribute new knowledge on validation methodology, and reveal important information about the indicators under study and the phenomena they target for monitoring. Validating the indicators in three diverse settings will explore the external validity of results.

Methods and analysis

This observational study explores the validity of ten indicators from the EPMM monitoring framework via seven discrete validation exercises that will utilize mixed methods: 1) cross-sectional review of policy data, 2) retrospective review of facility-level patient and administrative data, and 3) collection of primary quantitative and qualitative cross-sectional data from health service providers and clients. There is a specific methodological approach and analytic plan for each indicator, directed by unique, relevant validation research questions.

Ethics and dissemination

The protocol was approved by the Office of Human Research Administration at Harvard University in November, 2019. Individual study sites received approval via local institutional review boards by January 2020 except La Pampa, Argentina, approved June 2020.

Our dissemination plan enables unrestricted access and reuse of all published research, including data sets. We expect to publish at least one peer-reviewed publication per validation exercise. We will disseminate results at conferences, and engage local stakeholders in dissemination activities in each study country.

For peer review only - http://bmjopen.bmj.com/site/about/guidelines.xhtml

ARTICLE SUMMARY

Strengths and limitations of this study

- This research uses innovative methodological approaches to validate indicators for monitoring maternal health policy and maternal health system effectiveness, which are seldom systematically research-validated.
- The study scale addresses 10/25 of the metrics from the comprehensive monitoring framework for the "Strategies toward Ending Preventable Maternal Mortality (EPMM)" designed to monitor distal determinants of maternal mortality that comprise an enabling environment for maternal health and survival.
- The study methods target the underlying constructs that the ten discrete indicators are intended to measure and provide evidence to validate how well they reflect the phenomena they target for monitoring.
- Systematic sampling across twelve districts in three diverse settings increases the external validity of the results.
- The research does not reflect comprehensive national data but rather is limited to four subnational study settings in each country.

For peer review only - http://bmjopen.bmj.com/site/about/guidelines.xhtml

Word Count [4936/4000]

Introduction

Sustainable Development Goal (SDG) 3.1.1. targets a global maternal mortality ratio (MMR) of <70 maternal deaths per 100,000 live births by 2030. There were 295,000 maternal deaths in 2017, a global MMR of 211/100,000. If the average annual rate of reduction does not accelerate above 2.9%, the rate from 2000 to 2017, we will miss the target by 1 million preventable maternal deaths worldwide. (1) As countries move through the obstetric transition (2) and maternal deaths shift from direct obstetric to indirect causes, addressing upstream factors is critical to ending preventable maternal mortality. Graham et al. (2016) (3) illustrated the widening range of causes of death between and within countries. Thus, recognition is growing of the importance of social, political, economic, and structural factors that impact causes of death and health system responses to them. These include the status of women in societies, the functionality of health systems, access to universal health coverage and reproductive justice, the capacity to register all births and to count all deaths and track their causes, and to address all causes effectively. With acknowledgement of the significance of such distal determinants, improving metrics, data quality, and measurement capacity to monitor them has taken on greater urgency.

In 2015, the World Health Organization (WHO) released the "Strategies toward Ending Preventable Maternal Mortality (EPMM)" (EPMM Strategies) (4), a global guidance document outlining targets and strategies for reducing maternal mortality in the SDG period. Developed through extensive stakeholder consultations, the strategies address the broad spectrum of determinants of maternal health and survival, exemplified in 11 Key Themes.

Table 1. EPMM 11 Key Themes

**Guiding Principles**	1. Empower women, girls, families and communities
	2. Integrate maternal and newborn health, protect and support the mother-baby dyad
	3. Prioritize country ownership, leadership, and supportive legal, regulatory and financial frameworks
	4. Apply a human-rights framework to ensure that high-quality reproductive, maternal, and newborn health care is available, accessible and acceptable to all who need it
**Cross-cutting Actions**	5. Improve metrics, measurement systems, and data quality
	6. Prioritize adequate resources and effective health care financing
**Five Strategic Objectives**	7. Address inequities in access to and quality of sexual, reproductive, maternal and newborn healthcare
	8. Ensure universal health coverage for comprehensive sexual, reproductive, maternal, and newborn healthcare
	9. Address all causes of maternal mortality, reproductive and maternal morbidities and related disabilities
	10. Strengthen health systems to respond to the needs and priorities of women and girls
	11. Ensure accountability in order to improve quality of care and equity

For peer review only - http://bmjopen.bmj.com/site/about/guidelines.xhtml

In 2016, over 150 technical, policy, and country experts from 78 organizations worldwide participated in a five-round modified Delphi process to develop a comprehensive monitoring framework for the EPMM Strategies, comprising indicators centered on its 11 Key Themes. A set of 25 indicators, plus six indicator stratification factors to allow tracking of inequities and data transparency, were identified by participants as the strongest available measures for tracking progress toward the priority recommendations in the report. (5) The organizing framework of the EPMM 11 Key Themes and menu of associated indicators were designed to support national decision makers in identifying priority areas for improvement in their context, and in tracking and driving improvement in those areas deemed of greatest relevance and urgency.

Most efforts to assess maternal health indicator validity focus on measures of service coverage (6) (7) (8, 9) and, to a lesser extent, quality and reliability of service delivery (10) (11) (12). Fewer measures overall focus on the upstream enabling environment for maternal health care provision, and they are typically not subject to validation research. (13) Methods for validation of health system and policy-level indicators are therefore not well described.

Box 1. What is Indicator Validity?

WHAT DOES INDICATOR VALIDITY MEAN?

Validity asks, "Is this measurement truly representative of the concept under study?"

**Selected Types of Validity**	**Definition**
Content Validity	Does the indicator fully represent the content domain or concept to be measured?
Criterion-related Validity	How does the value of an indicator compare to an objective measure of truth?
Construct Validity	Do two indicators that are purported to measure the same construct "behave" in the same way?

In 2019, the WHO "Mother and Newborn Information for Tracking Outcomes and Results" (MoNITOR) expert working group commissioned a landscape analysis based on interviews with experts in maternal and newborn health (MNH) measurement to better understand how the field conceptualizes indicator validity, approaches to validation, and gaps in the science (11).

The analysis identified gaps in research on indicator validity conducted in LMIC settings, and poor knowledge translation about indicator validity to those settings. As a result, it found little application of information on validity in the evaluation and selection of indicators for national and subnational monitoring. Some types of indicators in particular lacked research-based validation, e.g. those for monitoring women's satisfaction and experiences of care; abortion services; as well as indicators derived from facility and routine data systems and the policy environment. Recommendations included engaging national stakeholders in discussions and research on indicator validity, and focusing beyond

For peer review only - http://bmjopen.bmj.com/site/about/guidelines.xhtml

diagnostic-style, criterion-related validity to encompass the meaningfulness of indicators, including the accurate definition of their underlying constructs and their utility to drive improvement.

Benova et al. (14) (2020) published a conceptual framework compiling definitions of indicator validity and approaches for assessing its various dimensions, based on interviews with practitioners of MNH measurement. The framework includes methodological approaches for assessing validity of indicators for tracking health policy and health system factors, and calls for more research in this domain.

To fill critical gaps in the assessment of maternal health measure validity, the present protocol describes multi-country research to be conducted in Argentina, Ghana, and India at both national and sub-national levels. The overall aim of the study is to improve maternal health measurement by validating ten indicators from the EPMM monitoring framework, in order to drive improvement and track progress towards achieving the priority recommendations outlined in the EPMM Strategies. Of note, this research assesses 40% of the indicators in the set of EPMM metrics designed to allow countries and global partners to monitor critical dimensions of the upstream enabling environment for maternal health. Furthermore, the indicators validated through this research reflect a broad range of these distal determinants, as they correspond to nine out of the 11 EPMM Key Themes (Figure 1).

Methods and analysis

This observational study explores the validity of ten indicators from the EPMM monitoring framework. It utilizes mixed methods, including 1) cross-sectional review of secondary policy, legal, and regulatory data, 2) retrospective review of facility-level patient and administrative data, and 3) collection of primary, quantitative, cross-sectional data from health service providers and clients. There is a specific methodological approach to validate each indicator. In two specific cases, two indicators designed to monitor a similar construct are compared to each other to explore their convergence and whether indicator adjustment could improve measure validity for that construct. These two indicator pairs share the same validation research questions and are studied in tandem. Thus, the validity of the ten EPMM indicators is evaluated via seven separate assessments, or validation exercises.

The ten EPMM indicators under study and the specific validation research questions for each indicator appear in Table 2. Nine indicators will be validated in all countries, and one additional indicator is to be validated in Ghana only due to local interest. Data collection began in January 2020, was suspended due to COVID-19, resumed May 2020, and is expected to be completed by November 2021 in all settings.

For peer review only - http://bmjopen.bmj.com/site/about/guidelines.xhtml

Table 2. Indicators for Validation and Validation Questions

Indicators for Validation	Validation Questions
1. Legal status of abortion	1. How does the law, as expressed in the national statute, compare to the Countdown indicator metadata and to the information available on the WHO Global Abortion Policies Project Database for the country?<br><br>2. Is there evidence that providers are consistently applying the law for each of the grounds on which abortion is legal?
2. If fees exist for health services in the public sector, are women of reproductive age (15-49) exempt from user fees for [maternal health -related] services	1. Does the free care law or policy in the country provide all of the categories of services included in the indicator free of charges or fees to users?<br><br>2. For the categories of services that should be free according to the law/policy in the country, is there evidence that women are paying user fees for them?<br><br>3. If evidence is found that demonstrates that women are paying for services that are supposed to be free according to the law/policy in the country, is there evidence that user fees are being levied in a systematically differential way to women?
3. Health worker density and distribution (per 1,000 population)<br><br>4. Density of midwives, by district (by births)<br><br>(*The validity of these two indicators designed to measure a related construct will be evaluated in tandem using the same research validation questions.)	1. How does the definition of a midwife/midwifery professional on record in the country compare to the ILO definition and to the ICM midwifery competencies?<br><br>2. What proportion of practicing midwives meet the ICM standard for competency as evidenced by an analysis of the tasks they have performed in the last 90-day period?

For peer review only - http://bmjopen.bmj.com/site/about/guidelines.xhtml

	3. How does the value of the estimate differ based on the denominator used?
5. Midwives are authorized to deliver basic emergency obstetric and newborn care	1. Does the national regulatory framework in country that authorizes midwives/MPs to deliver BEmONC match was has been reported for this indicator for all 7 signal functions?<br><br>2. For signal functions that midwives/MPs are authorized to perform according to national regulations, is there evidence they have performed these tasks in settings where EmONC is provided in last year?
6. Availability of functional EmOC facilities<br><br>7. Geographic distribution of facilities that provide basic and EmOC<br><br>(*The validity of these two indicators designed to measure a related construct will be evaluated in tandem using the same research validation questions.)	1. Is there evidence from facilities designated as B/CEmONC to demonstrate that they have performed all 7 signal functions in last 3 months as defined in the metadata for these indicators?<br><br>2.How does the value of the indicator differ based on the denominator used: 500,000 population/district vs. 20,000 birth/district vs. travel time (<2 hours for BEmONC)?
8. Maternal death review coverage	1. How does evidence from the facility level on maternal death reviews compare to the coverage of maternal death reviews reported at district level, through state or district reporting programs?<br><br>2. How does the number of facility deaths captured through review of facility patient register data compare to the number of deaths reported at the district level?<br><br>3. How does the value of the indicator reported compare to the value calculated using primary data?

For peer review only - http://bmjopen.bmj.com/site/about/guidelines.xhtml

9. Demand for family planning satisfied through modern methods of contraception	1. How does a direct measure of demand satisfaction for family planning (woman's self-report) compare to the assigned result provided by the DHS algorithm derived from the responses to the series of questions used to calculate the indicator (same woman surveyed) (construct validity)?<br><br>2. How does the value of the indicator vary based on a new data source/estimation method compared to an established source/method?
10. Presence of laws and regulations that guarantee women aged 15-49 access to sexual and reproductive health care, information, and education<br><br>(*Assessment of the validity of this indicator will be conducted using data from Ghana only due to local stakeholder interest.)	1. Do the laws or regulations as recorded on the national statute in Ghana match the definition of the indicator, fully including all 13 components? (Presence of laws)<br>2. How does the value of the indicator change using two different methods of computation (scoring)?

For peer review only - http://bmjopen.bmj.com/site/about/guidelines.xhtml

Research Settings

The research will be coordinated by a multi-country team of partners from all three countries and the US. Country partners were selected through a competitive process based on proposal strength and geographic diversity. One application was selected from Africa, Asia, and Latin America/Caribbean respectively, based on World Bank classification. (15)

The research will comprise national and sub-national data; however, fieldwork will be conducted in subnational settings in each country. Four districts/provinces in each country were selected for primary data collection. Sites were selected through a purposive, two-staged sampling approach based on a composite index of key maternal health indicators reflecting antepartum, intrapartum, and postpartum care coverage and MMR, used as a proxy of health system performance. First, one state/region in the highest-performing quartile of the index and one state/region in the lowest-performing quartile were selected. Second, one highest-performing district/province and one lowest-performing district/province were selected within each state/region. Due to low population density in Argentina, terciles were used. In addition, because there was almost no geographic variability in skilled birth attendance and early postnatal care coverage in data from Argentina where most births take place in facilities, Uterotonic Administration at Birth (UAB) was substituted in the index for this country.

Table 3. National and Subnational Research Settings

Country	State / Region	District/Province
Argentina	Centro	Buenos Aires
		La Pampa
	Noroeste	Salta
		Jujuy
Ghana	Brong Ahafo	Techiman North
		Sunyani Municipal
	Northern	Bunkpurugu-Yunyoo
		Tolon
India	Tamil Nadu	Thiruvallur
		Krishnagiri
	Uttar Pradesh	Meerut
		Gonda

Data Sources, Participants, and Sampling

Data required for validation vary by indicator; details of the data sources, participants, and sampling for each indicator are presented in Table 4.

For peer review only - http://bmjopen.bmj.com/site/about/guidelines.xhtml

Table 4. Data Sources, Participants, and Sampling Plan Detailed by Validation Exercise

Validation Exercise	National/Subnational Data Sources	Facility-Level Data			Individual-Level Data		
		Facility Selection	Facility Sampling Plan	Data Source	Participant Selection	Participant Sampling Plan	Data Source
**#1**	National/subnational document review<br><br>Countdown 2030 country profile<br><br>WHO Global Abortion Policies Project (GAPP) Database	Sample of Facilities within 20 PSUs<br><br>All higher-level facilities in study districts/provinces	All facilities that perform at least one maternal-health related service	No facility-level data collected	All health service providers who belong to professional cadres that are legally authorized to provide abortion within the study setting	All eligible health service providers in all eligible facilities	Survey administered to eligible providers
**#2**	National/subnational document review<br><br>WHO Maternal Newborn Child and Adolescent Health Policy Survey (MNCAH)	Sample of Facilities within 20 PSUs<br><br>All higher-level facilities in study districts/provinces	All facilities that perform at least one maternal-health related service	No facility-level data collected	Chief financial officer (or similar administrative position) for each facility<br><br>Woman who received maternal health-related services<br><br>Companion of choice (e.g. family member or friend, if applicable) for women who had a complicated	All chief financial officers in all eligible facilities<br><br>All eligible women (or their companion of choice) leaving eligible facilities	Interviews with chief financial officers<br><br>Exit interviews with women or their companion of choice

					birth and/or underwent a cesarean		
**#3a**	National/subnational document review<br><br>District/Provincial demographic data including total population, number of women of reproductive age, number of births, and number of pregnancies	Census of all facilities in study districts/provinces	All facilities that perform at least one maternal-health related service	Facility staff listing	All currently employed professionals who meet the International Labour Organization's description of midwifery professionals or midwifery associate professionals	All eligible providers in all eligible facilities (in facilities with more than 50 eligible providers, a random sample of 50 providers will be drawn).	Survey administered to midwifery professional/midwifery associate professionals
**#3b**	National/subnational document review	Sample of Facilities within 20 PSUs<br><br>All higher-level facilities in study districts/provinces	All C/B EmONC facilities	Not applicable	All currently employed professionals who meet the International Labour Organization's description of midwifery professionals or midwifery associate professionals	All eligible providers in all eligible facilities (in facilities with more than 50 eligible providers, a random sample of 50 providers will be drawn).	Survey administered to midwifery professional/midwifery associate professionals
**#4**	District/Provincial demographic data including total population, number of women of reproductive age,	Census of all facilities in study districts/provinces	All facilities that provide birth care in each district/province	Facility GIS locational data	Not Applicable	Not Applicable	Not Applicable

	number of births, and number of pregnancies						
**#5**	Health Information System Data<br><br>Death Reviews reported to district/province	Census of all facilities in study districts/provinces	All facilities that provide birth care in each district/province	Administrative data<br><br>Registers	Not Applicable	Not Applicable	Not Applicable
**#6**	Not Applicable	Not Applicable	Not Applicable	Not Applicable	Community-based Sample of women*	Women aged between 15 and 49 years in study districts	Individual interview
**#7**	National/subnational document review<br><br>United Nations 12th Inquiry Among Governments on Population and Development, Module II (Fertility, Family Planning, and Reproductive Health) Survey	Not Applicable	Not Applicable	Not Applicable	Not Applicable	Not Applicable	Not Applicable

For peer review only - http://bmjopen.bmj.com/site/about/guidelines.xhtml

In general, three types of data will be collected: policy/administrative, facility, and individual data.

Policy/Administrative Data

We will systematically search for national and subnational policies, laws, and regulations through a comprehensive desk review of relevant source documents in each country. Country research teams will consult with subject matter experts and data custodians to ensure all relevant documents were captured. Country-specific data will also be collected from global databases and repositories, as required by each indicator. Further, administrative and patient-level data will be collected from district/provincial-level health management information systems (HMIS).

Facility Data

Facilities will be selected based on data requirements for each indicator, using a multi-stage sampling plan (Figure 2). In the first stage, we will conduct a census of all public and private registered health facilities in each study district/province. For some indicators, data will be collected from all facilities in the census. Next, we will determine which maternal health-related services are provided at each facility in the census. We will collect information on provision of services within the five categories in the WHO Maternal Newborn Child and Adolescent Health (MNCAH) Policy Survey: 1) cesarean section, childbirth (normal delivery), delivery-related pharmaceutical products and medical supplies, 2) family planning, 3) antenatal care and insecticide treated bed nets, 4) postnatal care for mother, 5) testing and treatment for sexually transmitted infectious diseases, and cervical cancer screening. (16) Although infertility management is included in the WHO MNCAH Policy Survey, it is not in our study.

Thereafter, we will replicate the methodology used in Demographic and Health Surveys (DHS) (17) to define primary sampling units (PSUs), which are typically census tracts or discrete villages, depending on the country. We will randomly select 20 PSUs in each study district/province based on probability proportionate to size. Finally, we will define eligible facilities for each indicator within the sampled PSUs based on the services they provide relevant to the specific validation questions for that indicator. Eligible facilities for each indicator will include all lower-level primary health facilities within the PSUs that provide the relevant maternal health-related services, plus all higher-level facilities across the district/province.

Individual Data

Within study districts/provinces, we will collect primary, quantitative, individual-level data from study participants via surveys conducted at facilities and in communities. Eligible facility-based participants will include administrators; maternity care clinicians (midwives/midwifery professionals and clinical cadres legally authorized to provide induced abortions); women who received an included maternal-health related service at an eligible facility, and their chosen companions if they had a complicated childbirth or cesarean birth. Within eligible facilities, we will obtain a sample of staff participants as detailed in Table 4. We will enroll 1,040 women of reproductive age who received maternal health services in each country, representing 20 women per service/district for 260 women total per district.

Eligible community-based participants will include women of reproductive age (15-49 years). We will use the same 20 PSUs to obtain the community-based sample of women. Within each, a house listing exercise will identify households with women of reproductive age (15-49 years). From this list, 18 households per PSU will be randomly selected and 1420 women will be recruited, based on the

For peer review only - http://bmjopen.bmj.com/site/about/guidelines.xhtml

following sample size calculation: $n = \frac{Z^2 * pq}{d^2}$, where Z is the standard normal deviate, p is the proportion of population with characteristic, q is the proportion of population without characteristic, d is the degree of accuracy required. The sample size derived through this calculation (n=96) was further adjusted to reflect an estimated 10% non-response rate, a design effect of 2 to account for clustering, and a multiplier of 1.68 to account for the low prevalence of modern contraception in each country, yielding a final sample size of 355 women per district/province. Household surveys are infeasible in Argentina due to low population density, vast distances between households, and lack of cultural acceptance. Therefore, interviews will be conducted with a random sample of 360 women per district exiting from eligible facilities.

Eligibility and exclusion criteria

Facility eligibility criteria are detailed above. Participants will be considered eligible if they belong to one of the targeted participant groups listed above, and/or have received an included maternal health related-service, and meet the age of majority to consent or else provide assent along with parental consent if younger (less than 18 years old in Ghana and India; less than 16 years old in Argentina).

Exclusion criteria include not being proficient in the local language; not meeting the age of majority in the country, district, or province unless they can provide parental consent; being unable, unwilling, or lacking capacity to provide consent or assent.

Public and Patient Involvement

No patients were involved in the design, conducting, reporting, or dissemination of this study. We will engage local country stakeholders in a dissemination activity in each study country. We will disseminate results to district/provincial government units and participating health facilities as appropriate, to ensure that they can be used to drive progress and improvement in the study settings.

In the following section, we describe in detail the specific methodology and analytical plan for each indicator.

Indicator #1: Validating "legal status of abortion" as an indicator of equal access under the law

Aims: 1) To verify that the "legal status of abortion" indicator reported globally by each country accurately reflects the laws and statutes on record; and 2) To look for variation at the provider- and facility-level of the application of the legal categories under which abortion is lawful (legal grounds), and thus the accessibility of induced abortion.

Methods: This validation exercise will use mixed methods exploring two validation questions to test the global indicator on legal status of abortion. We will conduct a desk review of the legal grounds for induced abortion expressed in national laws (subnational laws, in Argentina), also capturing any requirements for eligibility on each legal ground articulated in the legal statutes. We will conduct surveys with health professionals whose scope of practice authorizes them to provide abortions services in each setting to explore provider knowledge of the legal grounds for abortion in their jurisdiction, and provider practices for determining patient eligibility on each legal ground, providing abortion services or referrals.

For peer review only - http://bmjopen.bmj.com/site/about/guidelines.xhtml

Analysis: For the first validation question, we will compare and describe any differences between legal statutes in each country, reported data in the Countdown indicator, and the WHO GAPP Database. For the second, we will tabulate the number of accurate survey responses among abortion providers on the legal grounds for abortion in their jurisdiction. We will explore any variance in provider requirements to access abortion for each legal ground in the country to look for differences in the application of the law across providers and facilities. Descriptive statistics will be reported and we will stratify the results to look for systematic variance.

Indicator #2: Validating reported policies on free maternal health-related services in the public sector

Aim: To verify that no charges, formal or informal, are assessed for services included in the indicator that are supposed to be free by law, and to describe variance between the law and primary data sources.

Methods: We will conduct a desk review of national and subnational laws and policies on free care provision. We will administer surveys to chief financial officers (or similar administrative position) within participating health facilities to collect data on formal fees or payments charged for any included services and the rationale. We will conduct interviews with women exiting eligible facilities to ask about formal and informal charges for any services received. If a woman had a complicated birth or cesarean section and a companion of choice (e.g. family member or friend) is present who was at the facility during the birth, we will interview them as well about any charges they may have paid on her behalf.

Analysis: We will use comparative analysis to detect and describe differences between service categories designated as free to users in the national statutes, and the most recent data reported by the country in the WHO MNCAH Policy Survey. We will estimate the percent of women paying fees for each type of service. Universal applicability of the policy implies that 0% of women pay fees for maternal health services in the public sector. We will test the significance in the difference using a one-sample test of proportion. We will use a chi-squared test to determine if fees are levied in a systematically different way to various types of women using the EPMM standard equity stratifiers. Results will be reported by service type and client demographics, and the value of the indicator expressed each way will be compared to explore optimal construct validity.

Indicators #3, # 4, and #5: Validating critical measures for monitoring adequacy of the midwifery workforce

Aim: To strengthen measurement of midwifery workforce adequacy. Three aspects of adequacy are reflected: density (number to meet need), distribution (accessibility), and both competency and authorization to provide essential care (availability).

Two nested validation exercises are included. The aims of the first one are: 1) to compare midwifery professionals' scope of practice in each country to international reference standards from the International Labour Organization's (ILO) definitions for midwifery professionals and associate professionals and to the International Confederation of Midwives (ICM) Essential Competencies for Midwifery Practice; and 2) to compare estimates derived from two indicators to measure the same construct (density and distribution of midwives), to explore consistency (convergent validity), evidence

For peer review only - http://bmjopen.bmj.com/site/about/guidelines.xhtml

that one measure is more accurate or a more efficient way to capture the construct, and whether adjusting the numerator and/or denominator provides a better estimate.

The second validation exercise aims to verify whether midwives and midwifery professionals are authorized to perform basic obstetric and neonatal care (BEmONC) functions, and whether they do so in practice.

Methods: We will conduct document review to compare the national scope of practice for midwifery professionals on record in each country to the ILO and ICM descriptions for midwifery personnel. We will review national laws and regulations that authorize midwifery professionals' scope of practice in each country to verify what is reported by the country in the MNCAH Policy Survey. Then, we will recruit a representative sample of midwifery professionals employed within all participating facilities providing maternal health-related services in each study district. We will administer a survey asking respondents whether they have the skills necessary to perform each competency and/or BEmONC signal function; how they obtained those skills; the frequency and recency of behaviors related to each competency; or reasons for non-performance of these behaviors in their current job.

Analysis: We will report the percent agreement between the national scope of midwifery practice and the ILO tasks, and the ICM competencies, and the variance between them. We will calculate the percent (%) of midwives whose current practice meets the international standard reflected in the ICM competencies as well as the average competency of midwives in the sample, stratified by facility type (public, private), and geography (urban, rural). Last, we will compare the value of the indicator for density and distribution of midwives, adjusted using different numerators and denominators. For numerators, we will calculate the value using the number of midwives on facility rosters, those who meet the ILO definition, and those who meet the ICM competencies. For the denominator, we will examine the value of the indicator using different population parameters: total population/district; women of reproductive age/district; number of births/district; and number of pregnancies/district.

We will compare midwives' authorization to perform BEmONC signal functions with the country's most recent Countdown 2030 country profile and responses to the most recent WHO RMNCH Policy Survey. We will then compare the tasks that midwives and midwifery professionals are authorized to perform to their reported actual performance of those tasks over the last 90-day period in facilities where emergency maternal and newborn care is available in each study setting. We will report any variance between midwifery professionals' authorization, training, and practice patterns.

Indicators # 6 and #7: Triangulating measures of availability - Validating indicators for monitoring "Availability of B/CEmoNC facilities"

Aim: To explore two dimensions of availability of B/CEmONC facilities: availability of all B/CEmONC signal functions within designated B/CEmONC facilities, and sufficient number of B/CEmONC facilities to meet the needs of the population (coverage). The aim is to compare the value of estimates emphasizing different dimensions of availability of B/CEmONC facilities, based on different measurement approaches and data sources, to explore their external consistency or convergent validity.

Methods: We will review records at all participating facilities where births take place to look for evidence that they have performed emergency signal functions within the previous 90 days and offer services 24 hours per day/7 days per week. We will perform geospatial analysis to estimate the travel

For peer review only - http://bmjopen.bmj.com/site/about/guidelines.xhtml

time to each facility within the sample for various segments of the population. We will use a publicly available global population model for these estimations.

Analysis: We will compare and report any variance between B/CEmONC designation and functionality across all facilities. We will calculate and compare the value of the indicator in each study district using the following denominators: 500,000 population/district; 20,000 births/district; 30,000 pregnancies/district. Last, we will use the travel time estimates obtained from the geospatial analysis to ascertain the number of facilities that are within a two-hour travel time for the total population, for women of reproductive age, and for the number of births and pregnancies occurring to women within each study district. We will explore how the value of the indicator differs based on the denominator used, and compare the values of the indicator reflecting these various approaches to measuring EmONC availability and report differences.

Indicator #8: Validating "maternal death review coverage" to improve maternal mortality data.

Aim: To validate both numerator and denominator of the indicator "Maternal death review coverage", defined as the percentage of maternal deaths occurring in a facility that were audited, in the study settings. Both numerator and denominator are subject to threats to validity due to under-reporting and misclassification of maternal deaths.

Methods: We will collect documentary evidence of maternal deaths and maternal deaths reviews in all facilities through chart and record review. We will perform retrospective review of secondary data obtained from district HMIS on both maternal deaths and maternal death reviews reported from all facilities.

Analysis: We will compare the number of facility-based maternal deaths reported through HMIS to the district to the verified number of maternal deaths in all facilities in the district in patient registers. We will trace individual deaths by dates and other reported details to verify they have been reported to the district. Once validated, we will aggregate all maternal deaths reported for comparison. We will review facility death review committee records for the last one-year period to extract the number of maternal death reviews conducted and the content of each review. We will compare the number of maternal death reviews reported to each district with the number of reviews validated through facility record review that met the definitional standard for quality (18) in the same district. Finally, we will tabulate maternal death review coverage using primary data for the numerator and denominator to the official value reported in the indicator in each country.

Indicator #9: Validating "Demand for family planning satisfied" from a woman-centered perspective: does the indicator reflect women's lived experience?

Aim: "Demand for family planning satisfied through modern methods of contraception" uses a macroeconomic lens to look at contraceptive supply and demand, aggregating data from individual women; however, it is uncertain how well it correlates with women's own subjective perceptions of their personal demand for contraception through modern methods or how well that demand has been satisfied. This study has two aims: 1) at the individual level, to assess whether women's self-reported demand for family planning and its satisfaction converges with the standard DHS-derived measure, and

For peer review only - http://bmjopen.bmj.com/site/about/guidelines.xhtml

2) at the population level, to examine how the value of the indicator changes based on the use of derived data from the standard calculation versus self-reported data reflecting women's own perceptions.

Methods: We will administer a community-based survey to a sample of women in each study setting that includes direct questions to women about their desire for and use of contraception, their satisfaction with their current method, and their experience of care during their most recent family planning encounter. We will then ask all the questions, in order, in the DHS algorithm used as the global standard to calculate the indicator.

Analysis: We will compare the results for individual women of two different approaches to measuring the construct of "demand for family planning satisfied through modern methods of contraception" using matched t-tests. We will disaggregate by women's characteristics to identify patterns. Finally, at the population level, we will compare the value of the indicator calculated from primary data we collect to the aggregate district/province level data reported through DHS where available to explore convergence.

Indicator #10: Comparative analysis of two scoring approaches to SDG 5.6.2. and their impact on the indicator value and interpretation of the results

Aim: Sustainable Development Goal 5.6.2. tracks the "Number of countries with laws and regulations that guarantee full and equal access to women and men aged 15 years and older to sexual and reproductive health care, information and education." Weaknesses with the indicator scoring methodology have the potential to change its value and affect its interpretation. The aim of this exercise is to verify the laws and regulations reported for this indicator in Ghana, and to explore whether the value of the indicator changes using new estimation methods to calculate its score compared to the established method, to improve interpretation.

Methods: We will conduct a comprehensive desk review of legal statutes and regulations related to the 13 components in the indicator metadata. We will conduct secondary analysis of results from the United Nations Twelfth Inquiry Among Governments on Population and Development, Module II (Fertility, Family Planning, and Reproductive Health) Survey(19), which reports on existing laws along with barriers and enablers.

Analysis: We will compare the laws and regulations on record in Ghana to the 13 components reported in the indicator for completeness and accuracy. We will calculate scores for the data collected from the UN Module II survey using the original UN scoring method and alternative scoring methods to look for differences in resulting values of the indicator. Values will be compared and sensitivity analyses conducted to explore the range of variation in the value of the indicator and the associated impact on its interpretation as a measure of sexual and reproductive health and rights.

Discussion

Because indicators for tracking maternal health system performance and effectiveness of maternal health policies rarely undergo systematic validation, methods for assessing such indicators are not codified. This research is expected to contribute new knowledge on validation methodology to the field of maternal health measurement.

For peer review only - http://bmjopen.bmj.com/site/about/guidelines.xhtml

Improving maternal health metrics, data quality, and measurement capacity is one of the eleven Key Themes highlighted in the EPMM Strategies. The results of this research will allow data custodians to strengthen core measures for monitoring a number of critical distal determinants of maternal mortality that comprise an enabling environment for maternal health and survival.

There are some limitations to the methodology proposed in this research protocol. We expect there will be data limitations. First, data will not be national. The scope of this research study is subnational, limited to four districts in two states within each of the three research country settings. Similarly, while a census of eligible health workers of various cadres is required to answer some of the validation questions to be explored in this research, we cannot oblige all members of the study population within the research settings to consent to participate in the study; we will attempt to address such limitations to the data we collect in the analysis.

Ethics and dissemination:

Ethical and safety considerations

All research partner organizations received approval to conduct human subjects research from each of their respective IRBs and obtained approvals or permissions as needed from their respective Ministry of Health and other required institutions.

Research staff in each country will obtain informed consent from participants prior to data collection. All potential participants in the study will be fully informed about the objectives, their right to refuse or to withdraw, and existing procedures for ensuring confidentiality. For participants below the age of majority (India and Ghana: 15-17 years old; Argentina: 15 years old) (Indicators 10, 22), written consent will be obtained from the parent or legal guardian of the minor, then written assent will be obtained from the minor. Both parties must consent to participate. Documentation of consent will occur after trained research staff have described the study and answered all outstanding questions. The participant and researcher will both sign and date the consent form. Participants who are illiterate will sign the form using their thumbprint. In the case of a self-administered electronic survey, consent may be obtained electronically from the participant prior to distribution of the electronic survey.

A data security plan is registered with the Institutional Review Board (IRB) of the Harvard T.H. Chan School of Public Health.

This study received approval from the following Ethical Review Boards:

USA: The Institutional Review Board (IRB) of the Harvard T.H. Chan School of Public Health, IRB 19-1086.
Argentina:

- La Secretaria de Coordinación General del Sistema de Salud-Dirección Provincial del Capital Humano-Comité Provincial de Ética de la Provincia de Jujuy
- El Ministerio de Salud Pública de Salta-Dirección de Recursos Humanos-Programa de docencia e investigación-Comisión provincial de investigaciones biomédicas-Comité de Ética de Investigación
- El Consejo de Bioética de la Provincia de La Pampa
- El Comité de Ética Central de la Provincia de Buenos Aires

Ghana: Ghana Health Service Ethics Review Committee, GHS-ERC022/08/19
India: Sigma IRB, 10052/IRB/19-20

For peer review only - http://bmjopen.bmj.com/site/about/guidelines.xhtml

Dissemination plan

Publication of the findings is planned through a special Collection in the PLoS Medicine journal. Data deposition will be in the Harvard Dataverse data repository per the Bill & Melinda Gates Foundation Open Access Policy.

Figure 1. Ten Indicators for Validation and their Corresponding EPMM Key Themes
Figure 2. Schematic of Standard Sampling Plan for Facilities

References

1. World Health O. Trends in maternal mortality 2000 to 2017: estimates by WHO, UNICEF, UNFPA, World Bank Group and the United Nations Population Division. Geneva: World Health Organization; 2019 2019.
2. Souza JP, Tunçalp Ö, Vogel J, Bohren M, Widmer M, Oladapo O, et al. Obstetric transition: the pathway towards ending preventable maternal deaths. BJOG: An International Journal of Obstetrics & Gynaecology. 2014;121:1-4.
3. Graham W, Woodd S, Byass P, Filippi V, Gon G, Virgo S. Maternal health 1 diversity and divergence: the dynamic burden of poor maternal health. Lancet. 2016;388:2164-75.
4. Organization WH. Strategies towards ending preventable maternal mortality (EPMM). 2015.
5. Jolivet RR, Moran AC, O'Connor M, Chou D, Bhardwaj N, Newby H, et al. Ending preventable maternal mortality: phase II of a multi-step process to develop a monitoring framework, 2016–2030. BMC pregnancy and childbirth. 2018;18(1):258.
6. Bryce J, Arnold F, Blanc A, Hancioglu A, Newby H, Requejo J, et al. Measuring coverage in MNCH: new findings, new strategies, and recommendations for action. PLoS Med. 2013;10(5):e1001423.
7. Munos MK, Stanton CK, Bryce J. Improving coverage measurement for reproductive, maternal, neonatal and child health: gaps and opportunities. Journal of global health. 2017;7(1).
8. Carvajal–Aguirre L, Vaz LM, Singh K, Sitrin D, Moran AC, Khan SM, et al. Measuring coverage of essential maternal and newborn care interventions: an unfinished agenda. Journal of global health. 2017;7(2).
9. Moran A, Marchant T. Measuring coverage of essential maternal and newborn care interventions: an unfinished agenda to define the data matrix for action in maternal and newborn health. Journal of global health. 2017;7(2).
10. Saturno-Hernández PJ, Martínez-Nicolás I, Moreno-Zegbe E, Fernández-Elorriaga M, Poblano-Verástegui O. Indicators for monitoring maternal and neonatal quality care: a systematic review. BMC pregnancy and childbirth. 2019;19(1):25.
11. Benova L, Moller A-B, Moran AC. "What gets measured better gets done better": The landscape of validation of global maternal and newborn health indicators through key informant interviews. PloS one. 2019;14(11):e0224746.
12. Moller A-B, Newby H, Hanson C, Morgan A, El Arifeen S, Chou D, et al. Measures matter: a scoping review of maternal and newborn indicators. PLoS One. 2018;13(10):e0204763.

For peer review only - http://bmjopen.bmj.com/site/about/guidelines.xhtml

13. George A, LeFevre AE, Jacobs T, Kinney M, Buse K, Chopra M, et al. Lenses and levels: the why, what and how of measuring health system drivers of women's, children's and adolescents' health with a governance focus. BMJ Global Health. 2019;4(Suppl 4):e001316.
14. Benova L, Moller A-B, Hill K, Vaz LM, Morgan A, Hanson C, et al. What is meant by validity in maternal and newborn health measurement? A conceptual framework for understanding indicator validation. PloS one. 2020;15(5):e0233969.
15. Bank TW. The World by Income and Region: The World Bank; [Available from: https://datatopics.worldbank.org/world-development-indicators/the-world-by-income-and-region.html.
16. Organization WH. Sexual, reproductive, maternal, newborn, child and adolescent health: policy survey, 2018-2019: summary report. 2020.
17. Corsi DJ, Neuman M, Finlay JE, Subramanian S. Demographic and health surveys: a profile. International journal of epidemiology. 2012;41(6):1602-13.
18. Organization WH. Time to respond: A report on the global implementation of maternal death surveillance and response. Geneva: WHO; 2016. 2017.
19. Lavrakas P. Encyclopedia of Survey Research Methods. 2008.

Authors contributions

1. Rima Jolivet and Jewel Gausman drafted this paper with inputs from all co-authors.
2. Rima Jolivet developed the proposal for funding with inputs from Ana Langer and colleagues from the EPMM Working Group and the Harvard T.H. Chan School of Public Health.
3. All co-authors collaborated to conceptualize and co-develop the research aims and methods. Jewel Gausman led the development of the analytic plans with review and input from all co-authors.
4. All co-authors provided substantive review feedback to finalize the paper.

Acknowledgements
Lenka Benova
Ann-Beth Moller
Allisyn Moran
Jeff Blossom

Funding statement
This work was supported by the Bill and Melinda Gates Foundation with grant number OPP1169546. Under the grant conditions of the Foundation, a Creative Commons Attribution 4.0 Generic License has already been assigned to the Author Accepted Manuscript version that might arise from this submission.

Availability of data and materials
No data were generated or analyzed for this study protocol; all relevant materials are included in this published article (and its supplementary information files.

This work is licensed under the Creative Commons Attribution 3.0 Unported License. To view a copy of this license, visit http://creativecommons.org/licenses/by/3.0/ or send a letter to Creative Commons, PO Box 1866, Mountain View, CA 94042, USA.

For peer review only - http://bmjopen.bmj.com/site/about/guidelines.xhtml

Competing interests statement
None declared.

DRAFT
For peer review only

For peer review only - http://bmjopen.bmj.com/site/about/guidelines.xhtml

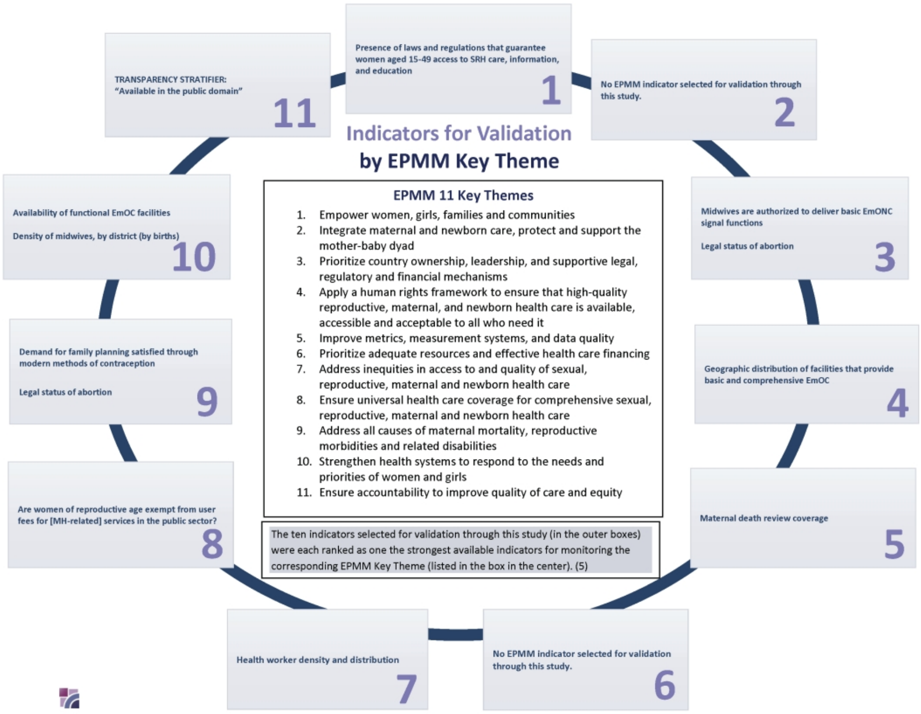

Figure 1. Ten Indicators for Validation and their Corresponding EPMM Key Themes

279x215mm (300 x 300 DPI)

Figure 2. Schematic of Standard Sampling Plan for Facilities

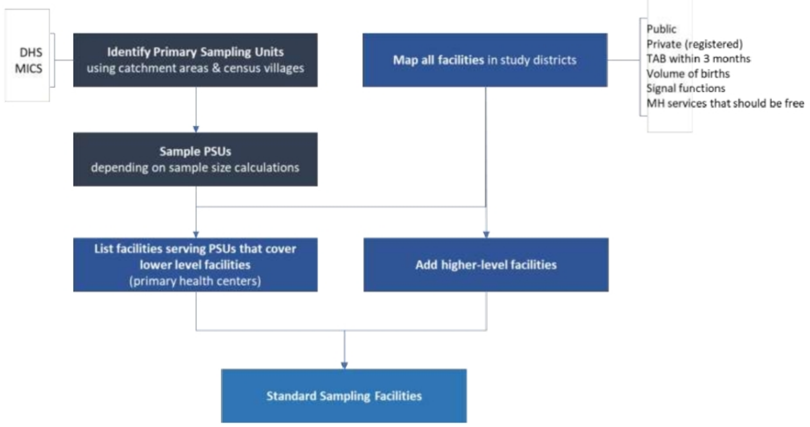

Figure 2. Schematic of Standard Sampling Plan for Facilities

279x215mm (200 x 200 DPI)

BMJ Open

A multisite, mixed methods study to validate ten maternal health system and policy indicators in Argentina, Ghana, and India: a research protocol

Journal:	*BMJ Open*
Manuscript ID	bmjopen-2021-049685.R2
Article Type:	Protocol
Date Submitted by the Author:	16-Dec-2021
Complete List of Authors:	Jolivet, R. Rima; Harvard University T H Chan School of Public Health, Global Health & Population<br>Gausman, Jewel; Harvard University T H Chan School of Public Health, Women and Health Initiative; Department of Global Health and Population<br>Adanu, Richard; University of Ghana School of Public Health,<br>Bandoh, Delia; University of Ghana School of Public Health<br>Belizan, M; Institute for Clinical Effectiveness and Health Policy<br>Bergel, Eduardo; Institute for Clinical Effectiveness and Health Policy<br>Berrueta, Mabel; Institute for Clinical Effectiveness and Health Policy, Data Center<br>Chakraborty, Suchandrima; Population Council<br>Kenu, Ernest; University of Ghana School of Public Health<br>Khan, Nizamuddin; Population Council India,<br>Odikro, Magdalene; University of Ghana<br>Pingray, Veronica; Institute for Clinical Effectiveness and Health Policy<br>Ramesh, Sowmya ; Population Council<br>Saggurti, Niranjan; Population Council<br>Vázquez, Paula; Institute for Clinical Effectiveness and Health Policy<br>Langer , Ana ; Harvard University Department of Global Health and Population
**Primary Subject Heading**:	Global health
Secondary Subject Heading:	Obstetrics and gynaecology, Public health, Health services research, Health policy
Keywords:	Health policy < HEALTH SERVICES ADMINISTRATION & MANAGEMENT, International health services < HEALTH SERVICES ADMINISTRATION & MANAGEMENT, OBSTETRICS, GYNAECOLOGY, PUBLIC HEALTH, REPRODUCTIVE MEDICINE

SCHOLARONE™
Manuscripts

Title: A multisite, mixed methods study to validate ten maternal health system and policy indicators in Argentina, Ghana, and India: a research protocol

Authors: R. Rima Jolivet[1], Jewel Gausman[1], Richard Adanu[2], Delia Bandoh[2], Maria Belizan[3], Mabel Berrueta[3], Suchandrima Chakraborty[4], Ernest Kenu[2], Nizamuddin Khan[4], Magdalene Odikro[2], Veronica Pingray[3], Sowmya Ramesh[4], Niranjan Saggurti[4], Paula Vázquez[3], Ana Langer[1]

1. Women and Health Initiative, Department of Global Health and Population, Harvard T.H. Chan School of Public Health, Boston, MA, USA
2. School of Public Health, University of Ghana, Legon, Accra,Ghana
3. Institute for Clinical Effectiveness and Health Policy (IECS), Buenos Aires, Argentina
4. Population Council, Inc., New Delhi, India

Corresponding Author:
R. Rima Jolivet, CNM, MSN, DrPH
Department of Global Health and Population
Harvard T.H. Chan School of Public Health
651 Huntington Avenue, FXB 643C, Boston, MA 02115
rjolivet@hsph.harvard.edu

Notes:

- The dates of the study are May 2020 – November 2021.

DRAFT
For peer review only

ABSTRACT [296/300]

Introduction

Most efforts to assess maternal health indicator validity focus on measures of service coverage. Fewer measures focus on the upstream enabling environment, and such measures are typically not research-validated. Thus, methods for validating system and policy-level indicators are not well described. This protocol describes original multi-country research to be conducted in Argentina, Ghana, and India, to validate ten indicators from the monitoring framework for the "Strategies toward Ending Preventable Maternal Mortality" (EPMM). The overall aim is to improve capacity to drive and track progress towards achieving the priority recommendations in the EPMM Strategies. This work is expected to contribute new knowledge on validation methodology, and reveal important information about the indicators under study and the phenomena they target for monitoring. Validating the indicators in three diverse settings will explore the external validity of results.

Methods and analysis

This observational study explores the validity of ten indicators from the EPMM monitoring framework via seven discrete validation exercises that will utilize mixed methods: 1) cross-sectional review of policy data, 2) retrospective review of facility-level patient and administrative data, and 3) collection of primary quantitative and qualitative cross-sectional data from health service providers and clients. There is a specific methodological approach and analytic plan for each indicator, directed by unique, relevant validation research questions.

Ethics and dissemination

The protocol was approved by the Office of Human Research Administration at Harvard University in November, 2019. Individual study sites received approval via local institutional review boards by January 2020 except La Pampa, Argentina, approved June 2020.

Our dissemination plan enables unrestricted access and reuse of all published research, including data sets. We expect to publish at least one peer-reviewed publication per validation exercise. We will disseminate results at conferences, and engage local stakeholders in dissemination activities in each study country.

For peer review only - http://bmjopen.bmj.com/site/about/guidelines.xhtml

ARTICLE SUMMARY

Strengths and limitations of this study

- This research uses innovative methodological approaches to validate indicators for monitoring maternal health policy and maternal health system effectiveness, which are seldom systematically research-validated.
- The study scale addresses 10/25 of the metrics from the comprehensive monitoring framework for the "Strategies toward Ending Preventable Maternal Mortality (EPMM)" designed to monitor distal determinants of maternal mortality that comprise an enabling environment for maternal health and survival.
- The study methods target the underlying constructs that the ten discrete indicators are intended to measure and provide evidence to validate how well they reflect the phenomena they target for monitoring.
- Systematic sampling across twelve districts in three diverse settings increases the external validity of the results.
- The research does not reflect comprehensive national data but rather is limited to four subnational study settings in each country.

DRAFT For peer review only

Word Count [4936/4000]

Introduction

Sustainable Development Goal (SDG) 3.1.1. targets a global maternal mortality ratio (MMR) of <70 maternal deaths per 100,000 live births by 2030. There were 295,000 maternal deaths in 2017, a global MMR of 211/100,000. If the average annual rate of reduction does not accelerate above 2.9%, the rate from 2000 to 2017, we will miss the target by 1 million preventable maternal deaths worldwide. (1) As countries move through the obstetric transition (2) and maternal deaths shift from direct obstetric to indirect causes, addressing upstream factors is critical to ending preventable maternal mortality. Graham et al. (2016) (3) illustrated the widening range of causes of death between and within countries. Thus, recognition is growing of the importance of social, political, economic, and structural factors that impact causes of death and health system responses to them. These include the status of women in societies, the functionality of health systems, access to universal health coverage and reproductive justice, the capacity to register all births and to count all deaths and track their causes, and to address all causes effectively. With acknowledgement of the significance of such distal determinants, improving metrics, data quality, and measurement capacity to monitor them has taken on greater urgency.

In 2015, the World Health Organization (WHO) released the "Strategies toward Ending Preventable Maternal Mortality (EPMM)" (EPMM Strategies) (4), a global guidance document outlining targets and strategies for reducing maternal mortality in the SDG period. Developed through extensive stakeholder consultations, the strategies address the broad spectrum of determinants of maternal health and survival, exemplified in 11 Key Themes.

Table 1. EPMM 11 Key Themes

**Guiding Principles**	1. Empower women, girls, families and communities
	2. Integrate maternal and newborn health, protect and support the mother-baby dyad
	3. Prioritize country ownership, leadership, and supportive legal, regulatory and financial frameworks
	4. Apply a human-rights framework to ensure that high-quality reproductive, maternal, and newborn health care is available, accessible and acceptable to all who need it
**Cross-cutting Actions**	5. Improve metrics, measurement systems, and data quality
	6. Prioritize adequate resources and effective health care financing
**Five Strategic Objectives**	7. Address inequities in access to and quality of sexual, reproductive, maternal and newborn healthcare
	8. Ensure universal health coverage for comprehensive sexual, reproductive, maternal, and newborn healthcare
	9. Address all causes of maternal mortality, reproductive and maternal morbidities and related disabilities
	10. Strengthen health systems to respond to the needs and priorities of women and girls
	11. Ensure accountability in order to improve quality of care and equity

For peer review only - http://bmjopen.bmj.com/site/about/guidelines.xhtml

In 2016, over 150 technical, policy, and country experts from 78 organizations worldwide participated in a five-round modified Delphi process to develop a comprehensive monitoring framework for the EPMM Strategies, comprising indicators centered on its 11 Key Themes. A set of 25 indicators, plus six indicator stratification factors to allow tracking of inequities and data transparency, were identified by participants as the strongest available measures for tracking progress toward the priority recommendations in the report. (5) The organizing framework of the EPMM 11 Key Themes and menu of associated indicators were designed to support national decision makers in identifying priority areas for improvement in their context, and in tracking and driving improvement in those areas deemed of greatest relevance and urgency.

Most efforts to assess maternal health indicator validity focus on measures of service coverage (6) (7) (8, 9) and, to a lesser extent, quality and reliability of service delivery (10) (11) (12). Fewer measures overall focus on the upstream enabling environment for maternal health care provision, and they are typically not subject to validation research. (13) Methods for validation of health system and policy-level indicators are therefore not well described.

The analysis identified gaps in research on indicator validity conducted in LMIC settings, and poor knowledge translation about indicator validity to those settings. As a result, it found little application of information on validity in the evaluation and selection of indicators for national and subnational monitoring. Some types of indicators in particular lacked research-based validation, e.g. those for monitoring women's satisfaction and experiences of care; abortion services; as well as indicators derived from facility and routine data systems and the policy environment. Recommendations included engaging national stakeholders in discussions and research on indicator validity, and focusing beyond diagnostic-style, criterion-related validity to encompass the meaningfulness of indicators, including the accurate definition of their underlying constructs and their utility to drive improvement.

Benova et al. (14) (2020) published a conceptual framework compiling definitions of indicator validity (Box 1) and approaches for assessing its various dimensions, based on interviews with practitioners of MNH measurement. The framework includes methodological approaches for assessing validity of indicators for tracking health policy and health system factors, and calls for more research in this domain. We used this framework to inform the development of our research methods, based on specific validation questions of relevance to each indicator undergoing assessment.

Box 1. What is Indicator Validity?

WHAT DOES INDICATOR VALIDITY MEAN?(14, 15)

Validity asks, "Is this measurement truly representative of the concept under study?"

**Selected Types of Validity**	**Definition**

For peer review only - http://bmjopen.bmj.com/site/about/guidelines.xhtml

Content Validity	Does the indicator fully represent the content domain or concept to be measured?
Criterion-related Validity	How does the value of an indicator compare to an objective measure of truth?
Construct Validity	Do two indicators that are purported to measure the same construct “behave” in the same way?

To fill critical gaps in the assessment of maternal health measure validity, the present protocol describes multi-country research to be conducted in Argentina, Ghana, and India at both national and sub-national levels. The overall aim of the study is to improve maternal health measurement by validating ten indicators from the EPMM monitoring framework, in order to drive improvement and track progress towards achieving the priority recommendations outlined in the EPMM Strategies. Of note, this research assesses 40% of the indicators in the set of EPMM metrics designed to allow countries and global partners to monitor critical dimensions of the upstream enabling environment for maternal health. Furthermore, the indicators validated through this research reflect a broad range of these distal determinants, as they correspond to nine out of the 11 EPMM Key Themes (Figure 1).

Methods and analysis

This observational study explores the validity of ten indicators from the EPMM monitoring framework. It utilizes mixed methods, including 1) cross-sectional review of secondary policy, legal, and regulatory data, 2) retrospective review of facility-level patient and administrative data, and 3) collection of primary, quantitative, cross-sectional data from health service providers and clients. Standard approaches for assessing the validity of policy and health system indicators are not available; therefore, we developed a specific methodological approach to validate each indicator, tailored to test the validation questions that reflect the specific aims and research questions relevant to each indicator undergoing validation and its underlying construct. Because there is no standard approach (metric or framework) for assessing validity of indicators of upstream health system functionality, we have developed a tailored analytical plan with appropriate statistics to compare the values of the reported indicators to evidence collected in each case. In two specific cases, two indicators designed to monitor a similar construct are compared to each other to explore their convergence and whether indicator adjustment could improve measure validity for that construct. These two indicator pairs share the same validation research questions and are studied in tandem. Thus, the validity of the ten EPMM indicators is evaluated via seven separate assessments, or validation exercises.

The ten EPMM indicators under study and the specific validation research questions for each indicator appear in Table 2. Nine indicators will be validated in all countries, and one additional indicator is to be validated in Ghana only due to local interest. Data collection began in January 2020, was suspended due to COVID-19, resumed May 2020, and is expected to be completed by November 2021 in all settings.

For peer review only - http://bmjopen.bmj.com/site/about/guidelines.xhtml

Table 2. Indicators for Validation and Validation Questions

Indicators for Validation	Validation Questions
1. Legal status of abortion	1. How does the law, as expressed in the national statute, compare to the Countdown indicator metadata and to the information available on the WHO Global Abortion Policies Project Database for the country? (Criterion validity)<br><br>2. Is there evidence that providers are consistently applying the law for each of the grounds on which abortion is legal? (Construct validity)
2. If fees exist for health services in the public sector, are women of reproductive age (15-49) exempt from user fees for [maternal health -related] services	1. Does the free care law or policy in the country provide all of the categories of services included in the indicator free of charges or fees to users? (Criterion validity)<br><br>2. For the categories of services that should be free according to the law/policy in the country, is there evidence that women are paying user fees for them? (Construct validity)<br><br>3. If evidence is found that demonstrates that women are paying for services that are supposed to be free according to the law/policy in the country, is there evidence that user fees are being levied in a systematically differential way to women? (Equity analysis)
3. Health worker density and distribution (per 1,000 population)<br><br>4. Density of midwives, by district (by births)<br><br>(*The validity of these two indicators designed to measure a related construct will be evaluated in tandem using the same research validation questions.)	1. How does the definition of a midwife/midwifery professional on record in the country compare to the ILO definition and to the ICM midwifery competencies? (Criterion validity)<br><br>2. What proportion of practicing midwives meet the ICM standard for competency as evidenced by an analysis of the tasks they have performed in the last 90-day period? (Construct validity)<br><br>3. How does the value of the estimate differ based on the denominator used? (Convergent validity)
5. Midwives are authorized to deliver basic emergency obstetric and newborn care	1. Does the national regulatory framework in country that authorizes midwives/MPs to deliver BEmONC match was has been reported for this indicator for all 7 signal functions? (Criterion validity)<br><br>2. For signal functions that midwives/MPs are authorized to perform according

For peer review only - http://bmjopen.bmj.com/site/about/guidelines.xhtml

	to national regulations, is there evidence they have performed these tasks in settings where EmONC is provided in last year? (Construct validity)
6. Availability of functional EmOC facilities<br><br>7. Geographic distribution of facilities that provide basic and EmOC<br><br>(*The validity of these two indicators designed to measure a related construct will be evaluated in tandem using the same research validation questions.)	1. Is there evidence from facilities designated as B/CEmONC to demonstrate that they have performed all 7 signal functions in last 3 months as defined in the metadata for these indicators? (Construct validity)<br><br>2.How does the value of the indicator differ based on the denominator used: 500,000 population/district vs. 20,000 birth/district vs. travel time (<2 hours for BEmONC)? (Convergent validity)
8. Maternal death review coverage	1. How does evidence from the facility level on maternal death reviews compare to the coverage of maternal death reviews reported at district level, through state or district reporting programs? (Criterion validity)<br><br>2. How does the number of facility deaths captured through review of facility patient register data compare to the number of deaths reported at the district level? (Convergent validity)<br><br>3. How does the value of the indicator reported compare to the value calculated using primary data? (Convergent validity)
9. Demand for family planning satisfied through modern methods of contraception	1. How does a direct measure of demand satisfaction for family planning (woman's self-report) compare to the assigned result provided by the DHS algorithm derived from the responses to the series of questions used to calculate the indicator (same woman surveyed) (Construct validity)?<br><br>2. How does the value of the indicator vary based on a new data source/estimation method compared to an established source/method? (Convergent validity)
10. Presence of laws and regulations that guarantee women aged 15-49 access to sexual and reproductive health care, information, and education<br><br>(*Assessment of the validity of this indicator will be conducted using data from Ghana only due to local stakeholder interest.)	1. Do the laws or regulations as recorded on the national statute in Ghana match the definition of the indicator, fully including all 13 components? (Presence of laws) (Criterion validity)<br>2. How does the value of the indicator change using two different methods of computation (scoring)? (Convergent validity)

For peer review only - http://bmjopen.bmj.com/site/about/guidelines.xhtml

Research Settings
The research will be coordinated by a multi-country team of partners from all three countries and the US. Country partners were selected through a competitive process based on proposal strength and geographic diversity. One application was selected from Africa, Asia, and Latin America/Caribbean respectively, based on World Bank classification. (16)

The research will comprise national and sub-national data; however, fieldwork will be conducted in subnational settings in each country. Four districts/provinces in each country were selected for primary data collection. Sites were selected through a purposive, two-staged sampling approach based on a composite index of key maternal health indicators reflecting antepartum, intrapartum, and postpartum care coverage and MMR, used as a proxy of health system performance. First, one state/region in the highest-performing quartile of the index and one state/region in the lowest-performing quartile were selected. Second, one highest-performing district/province and one lowest-performing district/province were selected within each state/region. In Argentina, some adjustments to the standard site selection protocol were implemented. Due to low population density, terciles were used. In addition, because there was almost no geographic variability in skilled birth attendance and early postnatal care coverage in data from Argentina where most births take place in facilities, Uterotonic Administration at Birth (UAB) was substituted in the index for this country. Finally, to avoid over-representation of data from Buenos Aires province due to its disproportionate size (total population of over 16.5 million), Region V of the province was selected in consultation with the National Ministry of Health to represent the province. Region V of Buenos Aires province comprises 13 counties, a total population of 3,432,962, 16 hospitals, and 319 primary health centers, and reflects similar sociodemographic, geographic, and health system characteristics as the entire province.

Table 3. National and Subnational Research Settings

Country	State / Region	District/Province
Argentina	Centro	Buenos Aires Region V
		La Pampa
	Noroeste	Salta
		Jujuy
Ghana	Brong Ahafo	Techiman North
		Sunyani Municipal
	Northern	Bunkpurugu-Yunyoo
		Tolon
India	Tamil Nadu	Thiruvallur
		Krishnagiri
	Uttar Pradesh	Meerut
		Gonda

Data Sources, Participants, and Sampling
Data required for validation vary by indicator; details of the data sources, participants, and sampling for each indicator are presented in Table 4.

For peer review only - http://bmjopen.bmj.com/site/about/guidelines.xhtml

Table 4. Data Sources, Participants, and Sampling Plan Detailed by Validation Exercise

Validation Exercise	National/Subnational Data Sources	Facility-Level Data			Individual-Level Data		
		Facility Selection	Facility Sampling Plan	Data Source	Participant Selection	Participant Sampling Plan	Data Source
**#1**	National/subnational document review<br><br>Countdown 2030 country profile<br><br>WHO Global Abortion Policies Project (GAPP) Database	Sample of Facilities within 20 PSUs<br><br>All higher-level facilities in study districts/provinces	All facilities that perform at least one maternal-health related service	No facility-level data collected	All health service providers who belong to professional cadres that are legally authorized to provide abortion within the study setting	All eligible health service providers in all eligible facilities	Survey administered to eligible providers
**#2**	National/subnational document review<br><br>WHO Maternal Newborn Child and Adolescent Health Policy Survey (MNCAH)	Sample of Facilities within 20 PSUs<br><br>All higher-level facilities in study districts/provinces	All facilities that perform at least one maternal-health related service	No facility-level data collected	Chief financial officer (or similar administrative position) for each facility<br><br>Woman who received maternal health-related services<br><br>Companion of choice (e.g. family member or friend, if applicable) for women who had a complicated birth and/or underwent a cesarean	All chief financial officers in all eligible facilities<br><br>All eligible women (or their companion of choice) leaving eligible facilities	Interviews with chief financial officers<br><br>Exit interviews with women or their companion of choice
**#3a**	National/subnational document review<br><br>District/Provincial demographic data including total population, number of women of reproductive age, number of births, and number of pregnancies	Census of all facilities in study districts/provinces	All facilities that perform at least one maternal-health related service	Facility staff listing	All currently employed professionals who meet the International Labour Organization's description of midwifery professionals or midwifery associate professionals	All eligible providers in all eligible facilities (in facilities with more than 50 eligible providers, a random sample of 50 providers will be drawn).	Survey administered to midwifery professional/midwifery associate professionals

For peer review only - http://bmjopen.bmj.com/site/about/guidelines.xhtml

**#3b**	National/subnational document review	Sample of Facilities within 20 PSUs<br><br>All higher-level facilities in study districts/provinces	All C/B EmONC facilities	Not applicable	All currently employed professionals who meet the International Labour Organization's description of midwifery professionals or midwifery associate professionals	All eligible providers in all eligible facilities (in facilities with more than 50 eligible providers, a random sample of 50 providers will be drawn).	Survey administered to midwifery professional/midwifery associate professionals
**#4**	District/Provincial demographic data including total population, number of women of reproductive age, number of births, and number of pregnancies	Census of all facilities in study districts/provinces	All facilities that provide birth care in each district/province	Facility GIS locational data	Not Applicable	Not Applicable	Not Applicable
**#5**	Health Information System Data<br><br>Death Reviews reported to district/province	Census of all facilities in study districts/provinces	All facilities that provide birth care in each district/province	Administrative data<br><br>Registers	Not Applicable	Not Applicable	Not Applicable
**#6**	Not Applicable	Not Applicable	Not Applicable	Not Applicable	Community-based Sample of women*	Women aged between 15 and 49 years in study districts	Individual interview
**#7**	National/subnational document review<br><br>United Nations 12th Inquiry Among Governments on Population and Development, Module II (Fertility, Family Planning, and Reproductive Health) Survey	Not Applicable	Not Applicable	Not Applicable	Not Applicable	Not Applicable	Not Applicable

For peer review only - http://bmjopen.bmj.com/site/about/guidelines.xhtml

In general, three types of data will be collected: policy/administrative, facility, and individual data.

Policy/Administrative Data

We will systematically search for national and subnational policies, laws, and regulations through a comprehensive desk review of relevant source documents in each country. Country research teams will consult with subject matter experts and data custodians to ensure all relevant documents were captured. Country-specific data will also be collected from global databases and repositories, as required by each indicator. Further, administrative and patient-level data will be collected from district/provincial-level health management information systems (HMIS).

Facility Data

Facilities will be selected based on data requirements for each indicator, using a multi-stage sampling plan (Figure 2). In the first stage, we will conduct a census of all public and private registered health facilities in each study district/province. For some indicators, data will be collected from all facilities in the census. Next, we will determine which maternal health-related services are provided at each facility in the census. We will collect information on provision of services within the five categories in the WHO Maternal Newborn Child and Adolescent Health (MNCAH) Policy Survey: 1) cesarean section, childbirth (normal delivery), delivery-related pharmaceutical products and medical supplies, 2) family planning, 3) antenatal care and insecticide treated bed nets, 4) postnatal care for mother, 5) testing and treatment for sexually transmitted infectious diseases, and cervical cancer screening. (17) Although infertility management is included in the WHO MNCAH Policy Survey, it is not in our study.

Thereafter, we will replicate the methodology used in Demographic and Health Surveys (DHS) (18) to define primary sampling units (PSUs), which are typically census tracts or discrete villages, depending on the country. We will randomly select 20 PSUs in each study district/province based on probability proportionate to size. Finally, we will define eligible facilities for each indicator within the sampled PSUs based on the services they provide relevant to the specific validation questions for that indicator. Eligible facilities for each indicator will include all lower-level primary health facilities within the PSUs that provide the relevant maternal health-related services, plus all higher-level facilities across the district/province.

Individual Data

Within study districts/provinces, we will collect primary, quantitative, individual-level data from study participants via surveys conducted at facilities and in communities. Eligible facility-based participants will include administrators; maternity care clinicians (midwives/midwifery professionals and clinical cadres legally authorized to provide induced abortions); women who received an included maternal-health related service at an eligible facility, and their chosen companions if they had a complicated childbirth or cesarean birth. Within eligible facilities, we will obtain a sample of staff participants as detailed in Table 4. We will enroll 1,040 women of reproductive age who received maternal health services in each country, representing 20 women per service/district for 260 women total per district.

Eligible community-based participants will include women of reproductive age (15-49 years). We will use the same 20 PSUs to obtain the community-based sample of women. Within each, a house listing exercise will identify households with women of reproductive age (15-49 years). From this list, 18 households per PSU will be randomly selected and 1420 women will be recruited, based on the

For peer review only - http://bmjopen.bmj.com/site/about/guidelines.xhtml

following sample size calculation: $n = \frac{Z^2 * pq}{d^2}$, where Z is the standard normal deviate, p is the proportion of population with characteristic, q is the proportion of population without characteristic, d is the degree of accuracy required. The sample size derived through this calculation (n=96) was further adjusted to reflect an estimated 10% non-response rate, a design effect of 2 to account for clustering, and a multiplier of 1.68 to account for the low prevalence of modern contraception in each country, yielding a final sample size of 355 women per district/province. Household surveys are infeasible in Argentina due to low population density, vast distances between households, and lack of cultural acceptance. Therefore, interviews will be conducted with a random sample of 360 women per district exiting from eligible facilities.

Eligibility and exclusion criteria

Facility eligibility criteria are detailed above. Participants will be considered eligible if they belong to one of the targeted participant groups listed above, and/or have received an included maternal health related-service, and meet the age of majority to consent or else provide assent along with parental consent if younger (less than 18 years old in Ghana and India; less than 16 years old in Argentina).

Exclusion criteria include not being proficient in the local language; not meeting the age of majority in the country, district, or province unless they can provide parental consent; being unable, unwilling, or lacking capacity to provide consent or assent.

Public and Patient Involvement

No patients were involved in the design, conducting, reporting, or dissemination of this study. We will engage local country stakeholders in a dissemination activity in each study country. We will disseminate results to district/provincial government units and participating health facilities as appropriate, to ensure that they can be used to drive progress and improvement in the study settings.

In the following section, we describe in detail the specific methodology and analytical plan for each indicator.

Indicator #1: Validating "legal status of abortion" as an indicator of equal access under the law

Aims: 1) To verify that the "legal status of abortion" indicator reported globally by each country accurately reflects the laws and statutes on record; and 2) To look for variation at the provider- and facility-level of the application of the legal categories under which abortion is lawful (legal grounds), and thus the accessibility of induced abortion.

Methods: This validation exercise will use mixed methods exploring two validation questions to test the global indicator on legal status of abortion. We will conduct a desk review of the legal grounds for induced abortion expressed in national laws (subnational laws, in Argentina), also capturing any requirements for eligibility on each legal ground articulated in the legal statutes. We will conduct surveys with health professionals whose scope of practice authorizes them to provide abortions services in each setting to explore provider knowledge of the legal grounds for abortion in their jurisdiction, and provider practices for determining patient eligibility on each legal ground, providing abortion services or referrals.

For peer review only - http://bmjopen.bmj.com/site/about/guidelines.xhtml

Analysis: For the first validation question, we will compare and describe any differences between legal statutes in each country, reported data in the Countdown indicator, and the WHO GAPP Database. For the second, we will tabulate the number of accurate survey responses among abortion providers on the legal grounds for abortion in their jurisdiction. We will explore any variance in provider requirements to access abortion for each legal ground in the country to look for differences in the application of the law across providers and facilities. Descriptive statistics will be reported and we will stratify the results to look for systematic variance.

Indicator #2: Validating reported policies on free maternal health-related services in the public sector

Aim: To verify that no charges, formal or informal, are assessed for services included in the indicator that are supposed to be free by law, and to describe variance between the law and primary data sources.

Methods: We will conduct a desk review of national and subnational laws and policies on free care provision. We will administer surveys to chief financial officers (or similar administrative position) within participating health facilities to collect data on formal fees or payments charged for any included services and the rationale. We will conduct interviews with women exiting eligible facilities to ask about formal and informal charges for any services received. If a woman had a complicated birth or cesarean section and a companion of choice (e.g. family member or friend) is present who was at the facility during the birth, we will interview them as well about any charges they may have paid on her behalf.

Analysis: We will use comparative analysis to detect and describe differences between service categories designated as free to users in the national statutes, and the most recent data reported by the country in the WHO MNCAH Policy Survey. We will estimate the percent of women paying fees for each type of service. Universal applicability of the policy implies that 0% of women pay fees for maternal health services in the public sector. We will test the significance in the difference using a one-sample test of proportion. We will use a chi-squared test to determine if fees are levied in a systematically different way to various types of women using the EPMM standard equity stratifiers. Results will be reported by service type and client demographics, and the value of the indicator expressed each way will be compared to explore optimal construct validity.

Indicators #3, # 4, and #5: Validating critical measures for monitoring adequacy of the midwifery workforce

Aim: To strengthen measurement of midwifery workforce adequacy. Three aspects of adequacy are reflected: density (number to meet need), distribution (accessibility), and both competency and authorization to provide essential care (availability).

Two nested validation exercises are included. The aims of the first one are: 1) to compare midwifery professionals' scope of practice in each country to international reference standards from the International Labour Organization's (ILO) definitions for midwifery professionals and associate professionals and to the International Confederation of Midwives (ICM) Essential Competencies for Midwifery Practice; and 2) to compare estimates derived from two indicators to measure the same construct (density and distribution of midwives), to explore consistency (convergent validity), evidence

For peer review only - http://bmjopen.bmj.com/site/about/guidelines.xhtml

that one measure is more accurate or a more efficient way to capture the construct, and whether adjusting the numerator and/or denominator provides a better estimate.

The second validation exercise aims to verify whether midwives and midwifery professionals are authorized to perform basic obstetric and neonatal care (BEmONC) functions, and whether they do so in practice.

Methods: We will conduct document review to compare the national scope of practice for midwifery professionals on record in each country to the ILO and ICM descriptions for midwifery personnel. We will review national laws and regulations that authorize midwifery professionals' scope of practice in each country to verify what is reported by the country in the MNCAH Policy Survey. Then, we will recruit a representative sample of midwifery professionals employed within all participating facilities providing maternal health-related services in each study district. We will administer a survey asking respondents whether they have the skills necessary to perform each competency and/or BEmONC signal function; how they obtained those skills; the frequency and recency of behaviors related to each competency; or reasons for non-performance of these behaviors in their current job.

Analysis: We will report the percent agreement between the national scope of midwifery practice and the ILO tasks, and the ICM competencies, and the variance between them. We will calculate the percent (%) of midwives whose current practice meets the international standard reflected in the ICM competencies as well as the average competency of midwives in the sample, stratified by facility type (public, private), and geography (urban, rural). Last, we will compare the value of the indicator for density and distribution of midwives, adjusted using different numerators and denominators. For numerators, we will calculate the value using the number of midwives on facility rosters, those who meet the ILO definition, and those who meet the ICM competencies. For the denominator, we will examine the value of the indicator using different population parameters: total population/district; women of reproductive age/district; number of births/district; and number of pregnancies/district.

We will compare midwives' authorization to perform BEmONC signal functions with the country's most recent Countdown 2030 country profile and responses to the most recent WHO RMNCH Policy Survey. We will then compare the tasks that midwives and midwifery professionals are authorized to perform to their reported actual performance of those tasks over the last 90-day period in facilities where emergency maternal and newborn care is available in each study setting. We will report any variance between midwifery professionals' authorization, training, and practice patterns.

Indicators # 6 and #7: Triangulating measures of availability - Validating indicators for monitoring "Availability of B/CEmoNC facilities"

Aim: To explore two dimensions of availability of B/CEmONC facilities: availability of all B/CEmONC signal functions within designated B/CEmONC facilities, and sufficient number of B/CEmONC facilities to meet the needs of the population (coverage). The aim is to compare the value of estimates emphasizing different dimensions of availability of B/CEmONC facilities, based on different measurement approaches and data sources, to explore their external consistency or convergent validity.

Methods: We will review records at all participating facilities where births take place to look for evidence that they have performed emergency signal functions within the previous 90 days and offer services 24 hours per day/7 days per week. We will perform geospatial analysis to estimate the travel

For peer review only - http://bmjopen.bmj.com/site/about/guidelines.xhtml

time to each facility within the sample for various segments of the population. We will use a publicly available global population model for these estimations.

Analysis: We will compare and report any variance between B/CEmONC designation and functionality across all facilities. We will calculate and compare the value of the indicator in each study district using the following denominators: 500,000 population/district; 20,000 births/district; 30,000 pregnancies/district. Last, we will use the travel time estimates obtained from the geospatial analysis to ascertain the number of facilities that are within a two-hour travel time for the total population, for women of reproductive age, and for the number of births and pregnancies occurring to women within each study district. We will explore how the value of the indicator differs based on the denominator used, and compare the values of the indicator reflecting these various approaches to measuring EmONC availability and report differences.

Indicator #8: Validating "maternal death review coverage" to improve maternal mortality data.

Aim: To validate both numerator and denominator of the indicator "Maternal death review coverage", defined as the percentage of maternal deaths occurring in a facility that were audited, in the study settings. Both numerator and denominator are subject to threats to validity due to under-reporting and misclassification of maternal deaths.

Methods: We will collect documentary evidence of maternal deaths and maternal deaths reviews in all facilities through chart and record review. We will perform retrospective review of secondary data obtained from district HMIS on both maternal deaths and maternal death reviews reported from all facilities.

Analysis: We will compare the number of facility-based maternal deaths reported through HMIS to the district to the verified number of maternal deaths in all facilities in the district in patient registers. We will trace individual deaths by dates and other reported details to verify they have been reported to the district. Once validated, we will aggregate all maternal deaths reported for comparison. We will review facility death review committee records for the last one-year period to extract the number of maternal death reviews conducted and the content of each review. We will compare the number of maternal death reviews reported to each district with the number of reviews validated through facility record review that met the definitional standard for quality (19) in the same district. Finally, we will tabulate maternal death review coverage using primary data for the numerator and denominator to the official value reported in the indicator in each country.

Indicator #9: Validating "Demand for family planning satisfied" from a woman-centered perspective: does the indicator reflect women's lived experience?

Aim: "Demand for family planning satisfied through modern methods of contraception" uses a macroeconomic lens to look at contraceptive supply and demand, aggregating data from individual women; however, it is uncertain how well it correlates with women's own subjective perceptions of their personal demand for contraception through modern methods or how well that demand has been satisfied. This study has two aims: 1) at the individual level, to assess whether women's self-reported demand for family planning and its satisfaction converges with the standard DHS-derived measure, and

For peer review only - http://bmjopen.bmj.com/site/about/guidelines.xhtml

2) at the population level, to examine how the value of the indicator changes based on the use of derived data from the standard calculation versus self-reported data reflecting women's own perceptions.

Methods: We will administer a community-based survey to a sample of women in each study setting that includes direct questions to women about their desire for and use of contraception, their satisfaction with their current method, and their experience of care during their most recent family planning encounter. We will then ask all the questions, in order, in the DHS algorithm used as the global standard to calculate the indicator.

Analysis: We will compare the results for individual women of two different approaches to measuring the construct of "demand for family planning satisfied through modern methods of contraception" using matched t-tests. We will disaggregate by women's characteristics to identify patterns. Finally, at the population level, we will compare the value of the indicator calculated from primary data we collect to the aggregate district/province level data reported through DHS where available to explore convergence.

Indicator #10: Comparative analysis of two scoring approaches to SDG 5.6.2. and their impact on the indicator value and interpretation of the results

Aim: Sustainable Development Goal 5.6.2. tracks the "Number of countries with laws and regulations that guarantee full and equal access to women and men aged 15 years and older to sexual and reproductive health care, information and education." Weaknesses with the indicator scoring methodology have the potential to change its value and affect its interpretation. The aim of this exercise is to verify the laws and regulations reported for this indicator in Ghana, and to explore whether the value of the indicator changes using new estimation methods to calculate its score compared to the established method, to improve interpretation.

Methods: We will conduct a comprehensive desk review of legal statutes and regulations related to the 13 components in the indicator metadata. We will conduct secondary analysis of results from the United Nations Twelfth Inquiry Among Governments on Population and Development, Module II (Fertility, Family Planning, and Reproductive Health) Survey(20), which reports on existing laws along with barriers and enablers.

Analysis: We will compare the laws and regulations on record in Ghana to the 13 components reported in the indicator for completeness and accuracy. We will calculate scores for the data collected from the UN Module II survey using the original UN scoring method and alternative scoring methods to look for differences in resulting values of the indicator. Values will be compared and sensitivity analyses conducted to explore the range of variation in the value of the indicator and the associated impact on its interpretation as a measure of sexual and reproductive health and rights.

Discussion
Because indicators for tracking maternal health system performance and effectiveness of maternal health policies rarely undergo systematic validation, methods for assessing such indicators are not codified. This research is expected to contribute new knowledge on validation methodology to the field of maternal health measurement.

For peer review only - http://bmjopen.bmj.com/site/about/guidelines.xhtml

Improving maternal health metrics, data quality, and measurement capacity is one of the eleven Key Themes highlighted in the EPMM Strategies. The results of this research will allow data custodians to strengthen core measures for monitoring a number of critical distal determinants of maternal mortality that comprise an enabling environment for maternal health and survival.

There are some limitations to the methodology proposed in this research protocol. We expect there will be data limitations. First, data will not be national. The scope of this research study is subnational, limited to four districts in two states within each of the three research country settings. Similarly, while a census of eligible health workers of various cadres is required to answer some of the validation questions to be explored in this research, we cannot oblige all members of the study population within the research settings to consent to participate in the study; we will attempt to address such limitations to the data we collect in the analysis.

Ethics and dissemination:

Ethical and safety considerations

All research partner organizations received approval to conduct human subjects research from each of their respective IRBs and obtained approvals or permissions as needed from their respective Ministry of Health and other required institutions.

Research staff in each country will obtain informed consent from participants prior to data collection. All potential participants in the study will be fully informed about the objectives, their right to refuse or to withdraw, and existing procedures for ensuring confidentiality. For participants below the age of majority (India and Ghana: 15-17 years old; Argentina: 15 years old) (Indicators 10, 22), written consent will be obtained from the parent or legal guardian of the minor, then written assent will be obtained from the minor. Both parties must consent to participate. Documentation of consent will occur after trained research staff have described the study and answered all outstanding questions. The participant and researcher will both sign and date the consent form. Participants who are illiterate will sign the form using their thumbprint. In the case of a self-administered electronic survey, consent may be obtained electronically from the participant prior to distribution of the electronic survey.

A data security plan is registered with the Institutional Review Board (IRB) of the Harvard T.H. Chan School of Public Health.

This study received approval from the following Ethical Review Boards:

USA: The Institutional Review Board (IRB) of the Harvard T.H. Chan School of Public Health, IRB 19-1086.
Argentina:

- La Secretaria de Coordinación General del Sistema de Salud-Dirección Provincial del Capital Humano-Comité Provincial de Ética de la Provincia de Jujuy
- El Ministerio de Salud Pública de Salta-Dirección de Recursos Humanos-Programa de docencia e investigación-Comisión provincial de investigaciones biomédicas-Comité de Ética de Investigación
- El Consejo de Bioética de la Provincia de La Pampa
- El Comité de Ética Central de la Provincia de Buenos Aires

Ghana: Ghana Health Service Ethics Review Committee, GHS-ERC022/08/19
India: Sigma IRB, 10052/IRB/19-20

For peer review only - http://bmjopen.bmj.com/site/about/guidelines.xhtml

Dissemination plan

Publication of the findings is planned through a special Collection in the PLoS Medicine journal. Data deposition will be in the Harvard Dataverse data repository per the Bill & Melinda Gates Foundation Open Access Policy.

Figure 1. Ten Indicators for Validation and their Corresponding EPMM Key Themes
Figure 2. Schematic of Standard Sampling Plan for Facilities

References

1. World Health O. Trends in maternal mortality 2000 to 2017: estimates by WHO, UNICEF, UNFPA, World Bank Group and the United Nations Population Division. Geneva: World Health Organization; 2019 2019.
2. Souza JP, Tunçalp Ö, Vogel J, Bohren M, Widmer M, Oladapo O, et al. Obstetric transition: the pathway towards ending preventable maternal deaths. BJOG: An International Journal of Obstetrics & Gynaecology. 2014;121:1-4.
3. Graham W, Woodd S, Byass P, Filippi V, Gon G, Virgo S. Maternal health 1 diversity and divergence: the dynamic burden of poor maternal health. Lancet. 2016;388:2164-75.
4. Organization WH. Strategies towards ending preventable maternal mortality (EPMM). 2015.
5. Jolivet RR, Moran AC, O'Connor M, Chou D, Bhardwaj N, Newby H, et al. Ending preventable maternal mortality: phase II of a multi-step process to develop a monitoring framework, 2016–2030. BMC pregnancy and childbirth. 2018;18(1):258.
6. Bryce J, Arnold F, Blanc A, Hancioglu A, Newby H, Requejo J, et al. Measuring coverage in MNCH: new findings, new strategies, and recommendations for action. PLoS Med. 2013;10(5):e1001423.
7. Munos MK, Stanton CK, Bryce J. Improving coverage measurement for reproductive, maternal, neonatal and child health: gaps and opportunities. Journal of global health. 2017;7(1).
8. Carvajal–Aguirre L, Vaz LM, Singh K, Sitrin D, Moran AC, Khan SM, et al. Measuring coverage of essential maternal and newborn care interventions: an unfinished agenda. Journal of global health. 2017;7(2).
9. Moran A, Marchant T. Measuring coverage of essential maternal and newborn care interventions: an unfinished agenda to define the data matrix for action in maternal and newborn health. Journal of global health. 2017;7(2).
10. Saturno-Hernández PJ, Martínez-Nicolás I, Moreno-Zegbe E, Fernández-Elorriaga M, Poblano-Verástegui O. Indicators for monitoring maternal and neonatal quality care: a systematic review. BMC pregnancy and childbirth. 2019;19(1):25.
11. Benova L, Moller A-B, Moran AC. "What gets measured better gets done better": The landscape of validation of global maternal and newborn health indicators through key informant interviews. PloS one. 2019;14(11):e0224746.
12. Moller A-B, Newby H, Hanson C, Morgan A, El Arifeen S, Chou D, et al. Measures matter: a scoping review of maternal and newborn indicators. PLoS One. 2018;13(10):e0204763.

For peer review only - http://bmjopen.bmj.com/site/about/guidelines.xhtml

13. George A, LeFevre AE, Jacobs T, Kinney M, Buse K, Chopra M, et al. Lenses and levels: the why, what and how of measuring health system drivers of women's, children's and adolescents' health with a governance focus. BMJ Global Health. 2019;4(Suppl 4):e001316.
14. Benova L, Moller A-B, Hill K, Vaz LM, Morgan A, Hanson C, et al. What is meant by validity in maternal and newborn health measurement? A conceptual framework for understanding indicator validation. PloS one. 2020;15(5):e0233969.
15. DeVellis RF. Scale development: Theory and applications: Sage; 2011.
16. Bank TW. The World by Income and Region: The World Bank; [Available from: https://datatopics.worldbank.org/world-development-indicators/the-world-by-income-and-region.html.
17. Organization WH. Sexual, reproductive, maternal, newborn, child and adolescent health: policy survey, 2018-2019: summary report. 2020.
18. Corsi DJ, Neuman M, Finlay JE, Subramanian S. Demographic and health surveys: a profile. International journal of epidemiology. 2012;41(6):1602-13.
19. Organization WH. Time to respond: A report on the global implementation of maternal death surveillance and response. Geneva: WHO; 2016. 2017.
20. Lavrakas P. Encyclopedia of Survey Research Methods. 2008.

Authors contributions

1. Rima Jolivet developed the proposal for funding with inputs from Ana Langer and colleagues from the global EPMM Working Group and the Harvard T.H. Chan School of Public Health.
2. All co-authors (RA, DB, MB, MB, SC, JG, RRJ, EK, NK, AL, MO, VP, SR, NS, PV) collaborated to conceptualize and co-develop the research aims and methods. Jewel Gausman led the development of the analytic plans with review and input from RA, DB, MB, MB, SC, EK, NK, AL, MO, VP, SR, NS, PV.
3. Rima Jolivet and Jewel Gausman drafted this paper with inputs from RA, DB, MB, MB, SC, EK, NK, AL, MO, VP, SR, NS, PV.
4. All co-authors (RA, DB, MB, MB, SC, JG, RRJ, EK, NK, AL, MO, VP, SR, NS, PV) provided substantive review feedback to finalize the paper.

Acknowledgements
Lenka Benova
Eduardo Bergel
Ann-Beth Moller
Allisyn Moran
Jeff Blossom

Funding statement
This work was supported by the Bill and Melinda Gates Foundation with grant number OPP1169546. Under the grant conditions of the Foundation, a Creative Commons Attribution 4.0 Generic License has already been assigned to the Author Accepted Manuscript version that might arise from this submission.

Availability of data and materials
No data were generated or analyzed for this study protocol; all relevant materials are included in this published article (and its supplementary information files).

For peer review only - http://bmjopen.bmj.com/site/about/guidelines.xhtml

This work is licensed under the Creative Commons Attribution 3.0 Unported License. To view a copy of this license, visit http://creativecommons.org/licenses/by/3.0/ or send a letter to Creative Commons, PO Box 1866, Mountain View, CA 94042, USA.

Competing interests statement

None declared.

DRAFT
For peer review only

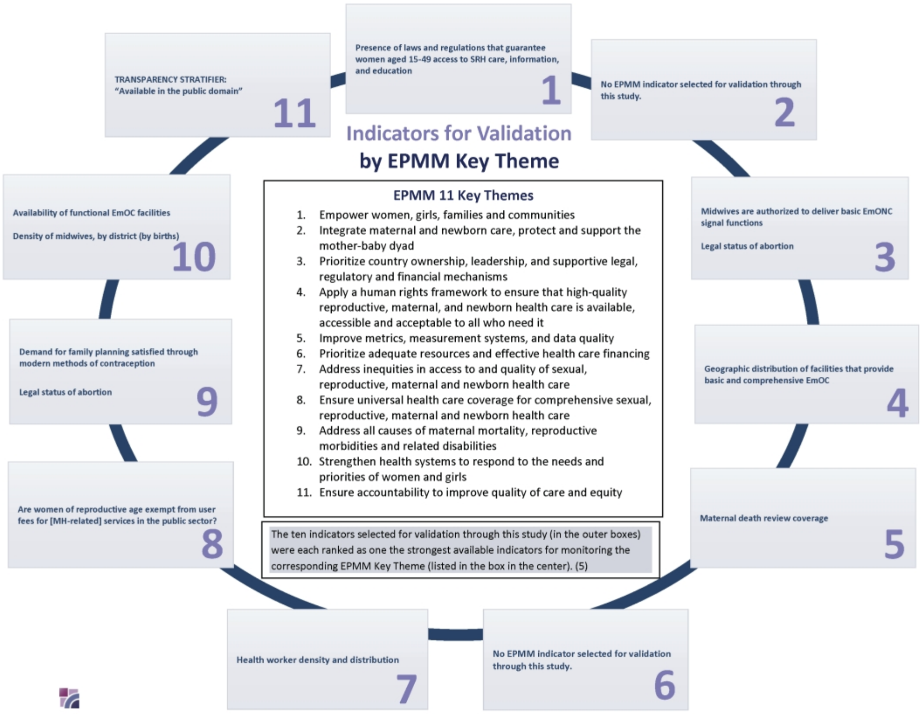

Figure 1. Ten Indicators for Validation and their Corresponding EPMM Key Themes

279x215mm (300 x 300 DPI)

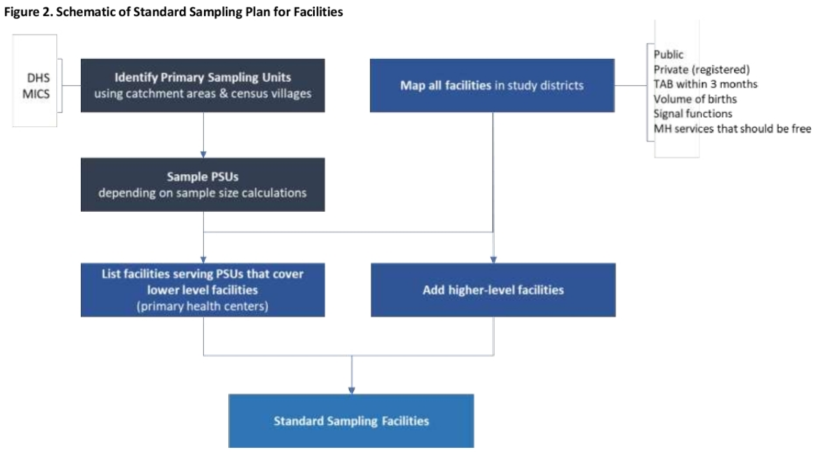

Figure 2. Schematic of Standard Sampling Plan for Facilities

279x215mm (200 x 200 DPI)
